# When high accuracy misleads in literature-derived machine learning for deep eutectic solvent recommendation

**DOI:** 10.1039/d6ra05453f

**Published:** 2026-08-04

**Authors:** Hakim Faraji, Juan A. Mendez-Perez, Ruth Rodríguez-Ramos, Bárbara Socas-Rodríguez, Antonio V. Herrera Herrera

**Affiliations:** a Departamento de Química, Área de Química Analítica, Facultad de Ciencias, Universidad de La Laguna (ULL) Avenida Astrofísico Francisco Sánchez s/n 38206 San Cristóbal de La Laguna Tenerife Spain hafaraji@ull.edu.es; b Departamento de Ingeniería Informática y de Sistemas, Universidad de La Laguna (ULL) Tenerife Spain; c CIMO, LA SusTEC, Instituto Politécnico de Bragança Campus de Santa Apolónia Bragança 5300-253 Portugal; d Instituto Universitario de Bio-Orgánica Antonio González, Universidad de La Laguna (ULL) Tenerife Spain

## Abstract

Deep eutectic solvents (DESs) are widely used in analytical sample preparation, yet selecting suitable systems for specific analytes remains challenging due to the large combinatorial design space and reliance on empirical screening. Machine learning (ML) has been proposed as a data-driven alternative, but its reliability under literature-derived data constraints is unclear. In this study, a literature-derived experiment-level dataset for DES-based pesticide extraction was reconstructed and evaluated under a leakage-controlled, DOI-grouped validation framework. After screening, cleaning, and descriptor eligibility filtering, the final modeling table comprised 757 records from 94 studies, organized into 626 (DOI, analyte) groups. Following a comprehensive leakage audit, the modeling pipeline was rebuilt using a split-first, training-only strategy with leakage-clean descriptors. The hybrid model, combining global classification and pairwise preference learning, improved the record-level classification performance (ROC-AUC, MCC) relative to the baseline, but did not provide a stable group-level ranking benefit under the present literature-derived data structure. In restricted comparable multi-candidate groups, ranking performance decreased and the hybrid model frequently underperformed, with no statistically significant advantage observed. Sanity baseline analysis further showed that the baseline model captured non-trivial structure beyond random and simple heuristic ranking in comparable groups, whereas the hybrid formulation did not provide any stable additional benefit. These results are explained by sparse comparative structure, study-bounded target definition, and limited descriptor representation. Overall, this work demonstrates that in literature-derived DES extraction settings, improvements in predictive accuracy do not necessarily translate into reliable recommendation capability, highlighting the need for data-centric evaluation, comparable candidate reporting, and structured experimental metadata for ML-driven chemical recommendations.

## Abstract 2

Leakage-clean ML improves classification for literature-derived DES data, but reliable recommendation requires comparable candidate sets and structured experimental reporting.

## Graphical abstract

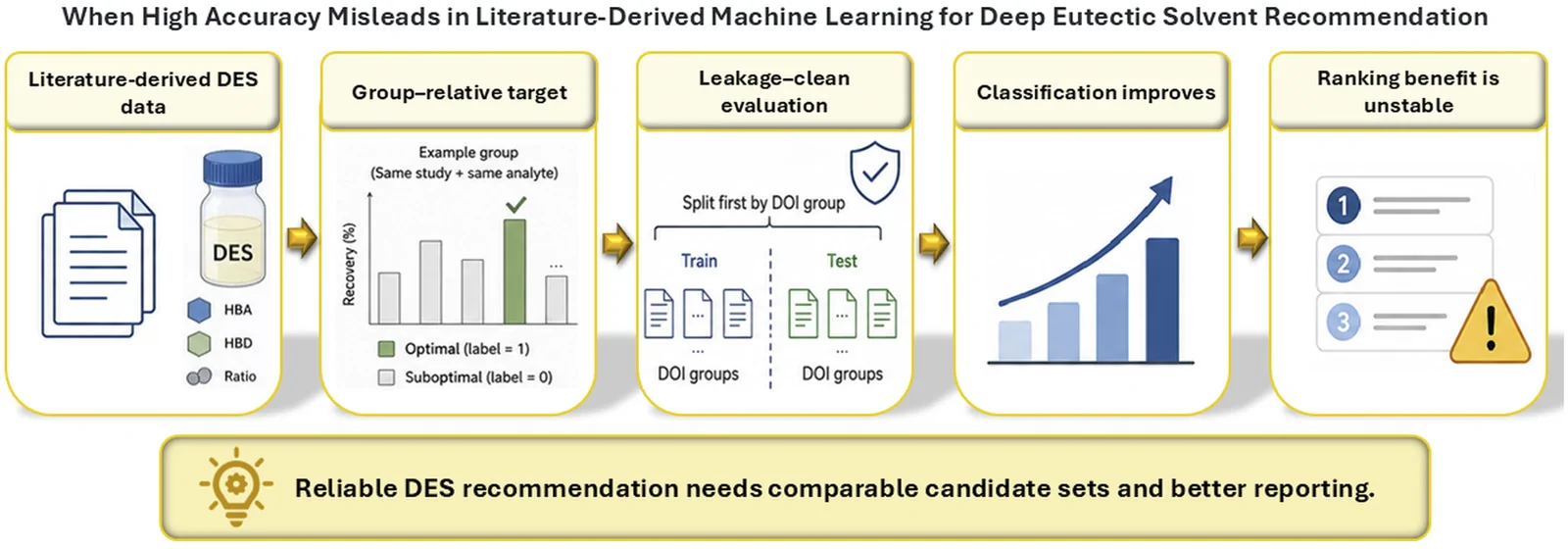

## 1 Introduction

Deep eutectic solvents (DESs) have emerged as versatile and environmentally favorable alternatives to conventional organic solvents in analytical chemistry, particularly in sample preparation and extraction workflows. Their tunable composition—defined by combinations of hydrogen bond acceptors (HBAs), hydrogen bond donors (HBDs), and molar ratios—enables control over key physicochemical properties such as polarity, viscosity, and hydrogen-bonding capacity. As a result, DES-based systems have been widely explored for the extraction of organic contaminants, including pesticides, across diverse matrices.^1,2^

Despite these advantages, selecting an appropriate DES system for a given analyte remains a non-trivial task. The design space is inherently combinatorial, yet experimental studies typically evaluate only a limited number of candidate systems, often focusing on structurally related DESs or narrow compositional variations.^3,4^ This constrained exploration limits coverage of the underlying chemical space and results in datasets that are sparse, highly structured, and often biased toward locally optimized solutions.

In response, machine learning (ML) approaches have been increasingly proposed to accelerate DES design and selection. Most existing studies focus on predicting physicochemical properties such as density, viscosity, or solubility, where relatively structured datasets are available.^5,6^ In contrast, the use of ML for recommending DES systems for specific analytical tasks—particularly extraction of target analytes—remains limited and conceptually more challenging.^7^ However, whether such approaches can support reliable recommendation under realistic, literature-derived data constraints remains an open question.

A key assumption underlying literature-driven ML approaches is that experimental data reported in published studies provide sufficient supervision for learning transferable relationships. However, literature-derived datasets differ fundamentally from designed datasets: they are generated through independent, study-specific optimization processes, typically aimed at identifying a single best-performing system rather than systematically characterizing performance across competing alternatives. As a consequence, such datasets may contain abundant information about clearly suboptimal conditions but limited information about relative differences among near-optimal candidates.

From a ML perspective, this leads to uneven and context-dependent supervision. While coarse discrimination between optimal and non-optimal systems may be learnable, the fine-grained comparisons required for recommendation—*i.e.*, ranking multiple candidates within the same decision context—may be weakly supported. In addition, experimental outcomes are inherently conditioned by study-specific factors such as matrix, extraction conditions, and candidate selection, which are not always consistently reported or fully represented in the available descriptors.^8,9^

An additional methodological concern is the potential for information leakage in literature-derived modeling pipelines.^10^ Variables derived from experimental outcomes—such as recovery, enrichment factor, or performance-based indicators—may inadvertently be incorporated into the feature space, leading to overly optimistic estimates of model performance. Pairwise or delta-based feature constructions are particularly susceptible to this issue if outcome-related information is not strictly excluded.^11^

Finally, evaluation of recommendation models requires careful alignment between metrics and the structure of the underlying data. Standard record-level metrics such as ROC-AUC or MCC assess global classification performance, but do not directly capture the ability of a model to make context-specific decisions among competing candidates. In recommendation settings, the relevant unit of evaluation is the set of alternatives defined within a given experimental context, rather than individual records considered in isolation.

In this work, we critically examine the feasibility of literature-driven ML for DES recommendation in analytical extraction. Rather than focusing on maximizing predictive performance, our objective is to assess whether the structure and informational content of literature-derived datasets support meaningful recommendation behavior. Specifically, we (i) reconstruct an experiment-level dataset from DES-based pesticide extraction studies, (ii) develop a leakage-aware modeling framework, and (iii) evaluate model behavior under group-consistent validation settings that reflect realistic experimental contexts.

Within the present literature-derived DES extraction setting, this study adopts a data-centric perspective, arguing that model performance depends not only on algorithmic design but also on how experimental data are generated, structured, and reported.^12^

## 2 Methodology

**Fig. 1:**
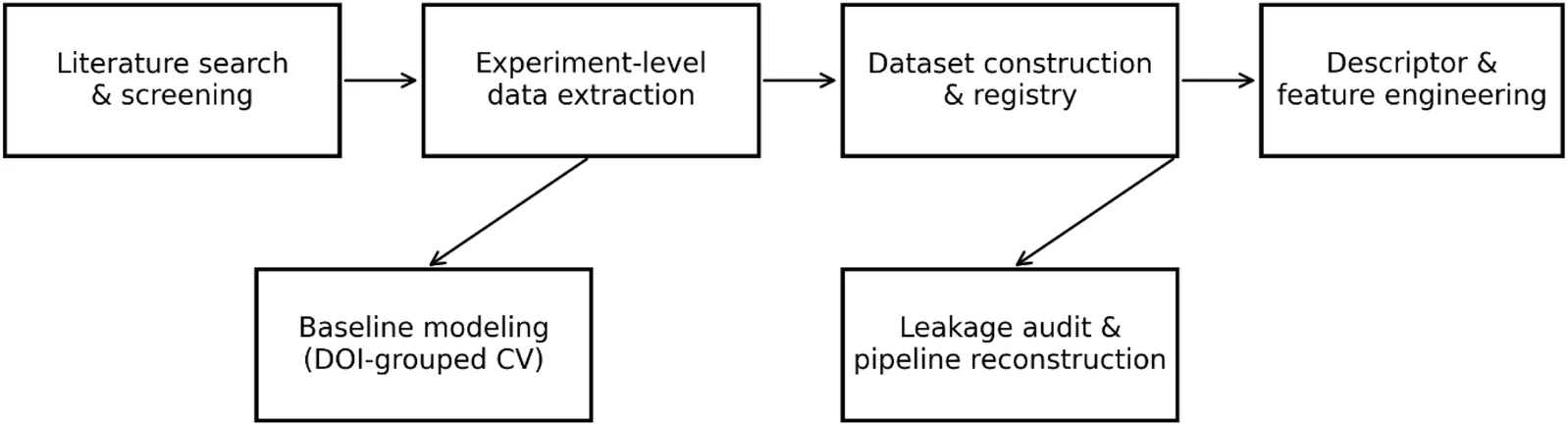
Overview of the data-driven workflow from literature extraction to leakage-clean model evaluation.

The overall workflow of the data-driven framework, from literature extraction to leakage-clean model evaluation, is summarized in Fig. 1.

### 2.1 Literature search and data extraction

A structured literature search was conducted in Scopus using the keywords “deep eutectic solvents pesticides” in article title, abstract, and keywords, limited to research articles. This search identified 207 records. Full texts were obtained for 197 articles and screened for relevance to DES-based pesticide determination or extraction. Articles containing DES and pesticide terminology but not reporting DES-based pesticide measurement or extraction data were excluded. After full-text relevance screening, 132 studies were retained for experiment-level extraction. A complete dataset-flow summary from literature search to final leakage-clean modeling table is provided in Table S18.

Experiment-level data were extracted using a structured AI-assisted workflow designed to capture all reported DES systems within each study, including optimal and suboptimal conditions, without aggregation or inference. Each DES-analyte-condition combination was treated as an independent record.

To assess extraction reliability, a random subset of 40 retained studies (approximately 30% of the studies retained for experiment-level extraction) was manually validated against the original publications and their SI files. Validation was performed at the field level for core fields affecting DES identity, analyte identity, grouping, target assignment, and descriptor construction. In total, 1200 fields were checked, and 46 discrepancies were identified prior to correction, corresponding to a pre-correction field-level accuracy of 96.2%. All identified discrepancies were corrected by manual verification against the source publications. Additional details on the validation procedure, error categories, representative corrected entries, and limitations of the audit are provided in the SI (Text S2 and Tables S1, S21, S22).

### 2.2 Dataset construction and target definition

The retained studies were processed using the structured extraction workflow, yielding 956 experiment-level records. After data cleaning, consistency filtering, chemical standardization, and descriptor eligibility filtering, the final modeling table contained 757 records from 94 source studies. All chemical components were standardized and mapped to canonical SMILES representations, and each DES system was defined as a unique combination of HBA, HBD, and molar ratio.

Experimental records were organized into competitive groups defined at the (DOI, analyte) level, such that each group contains all DES systems evaluated for a given analyte within a single study. This grouping ensures that comparisons are performed within a consistent experimental context.

The binary target variable, is_optimal, was defined within each group. DES systems achieving the maximum reported recovery within the same (DOI, analyte) group were labeled as optimal (is_optimal = 1), while all other systems were labeled as non-optimal (is_optimal = 0). In cases of ties, multiple systems were assigned the optimal label.

Accordingly, the target should not be interpreted as an intrinsic chemical optimum, but as the best observed condition within a study-specific candidate set. This target is therefore conditional on the experimental design, candidate coverage, and reported context of each source study. The learning problem is thus relative and study-bounded by construction. This formulation should be interpreted as an operational target definition for the present study rather than as a unique or universal representation of chemical optimality. Alternative target formulations, such as top-k labels, recovery-threshold labels, pairwise Δrecovery labels, or direct recovery prediction, may yield different model behavior. These alternatives were not used as primary targets because they address different prediction questions and require stronger assumptions about cross-study comparability of recovery values and experimental conditions. The present target was selected because it reflects the decision structure most consistently recoverable from the literature-derived screening data: identifying the best observed DES system within a study-specific candidate set. Because the target variable is defined conditionally within each (DOI, analyte) group, global feature-target correlation analysis was not considered sufficiently informative for the aims of this study and could be misleading outside the group context.^13^

### 2.3 Descriptor calculation and feature engineering

Molecular descriptors were computed from canonical SMILES representations using RDKit. A set of interpretable physicochemical descriptors was selected, including molecular weight, octanol–water partition coefficient (log *P*), topological polar surface area (TPSA), hydrogen-bond donor and acceptor counts, number of rotatable bonds, ring count, and heavy atom count.^14^

Descriptors were calculated for HBA, HBD, and analyte components, and subsequently combined to generate both DES-level aggregate descriptors and DES-analyte interaction features. DES-level descriptors were constructed using composition-weighted representations based on molar ratios, while interaction features were derived using difference, ratio, and product transformations to capture relative compatibility between DES systems and analytes.

The selected descriptors were chosen to represent chemically interpretable factors expected to influence DES-analyte compatibility. log *P*-related descriptors provide coarse proxies for hydrophobicity and polarity matching, which are relevant to partitioning of pesticide analytes into the DES phase. TPSA-related descriptors approximate polar surface compatibility, while hydrogen-bond donor and acceptor descriptors represent potential donor–acceptor complementarity between analytes and DES components. Molecular weight and heavy-atom descriptors provide approximate size and dispersion-related information, whereas rotatable-bond and ring-count descriptors capture simple conformational and structural features. Molar-ratio-weighted DES descriptors were used to approximate composition-dependent properties of the DES system, and DES-analyte difference, ratio, and product terms were included as simple leakage-clean proxies for physicochemical mismatch and interaction strength. These descriptors are not intended to fully represent the extraction mechanism, but to provide a low-dimensional and interpretable representation compatible with the information consistently available in literature-derived records.

The mathematical definitions of all engineered features and their physicochemical rationale are provided in the SI (Text S4 and Tables S2, S3, S23).

### 2.4 Limitation of the descriptor space

This feature space was intentionally restricted to leakage-clean, interpretable, descriptor-based variables. It does not explicitly encode several experimentally important determinants of extraction performance, such as matrix effects, pH, water content, ionic strength, or process-specific conditions, unless such information is indirectly reflected in the reported records.

### 2.5 Feature refinement and leakage control

To ensure valid model evaluation, strict leakage control was applied. All outcome-related variables, including recovery, enrichment factor, limit of detection (LOD), and any derived indicators, were excluded from the feature space prior to model training. The excluded variables and their rationale are summarized in Table 1.

**Table 1:** Leakage-prone variables excluded from the revised modeling space

Feature	Category	Reason for exclusion
all_parse_ok	Parsing/technical flag	Potential data-processing artifact; not chemically meaningful for prediction
analyte_parse_ok	Parsing/technical flag	Potential data-processing artifact; not chemically meaningful for prediction
cluster	*Post hoc* grouping variable	Not a valid pre-outcome chemical predictor
ef	Outcome-derived metric	Directly linked to experimental performance
hba_descriptor_eligible_phase1	Eligibility/technical flag	Technical preprocessing indicator, not a predictive chemical variable
hba_parse_ok	Parsing/technical flag	Potential data-processing artifact
hbd_descriptor_eligible_phase1	Eligibility/technical flag	Technical preprocessing indicator, not a predictive chemical variable
hbd_parse_ok	Parsing/technical flag	Potential data-processing artifact
is_optimal	Target-related label	Direct target leakage
lod	Outcome-derived metric	Directly linked to analytical performance
ratio_parse_ok	Parsing/technical flag	Potential data-processing artifact
recovery	Outcome-derived metric	Used in target definition; direct leakage
target	Target-related label	Direct target leakage
was_inferior	Outcome-derived flag	Derived from comparative performance

Additional preprocessing steps included removal of features with high missingness, near-zero variance, and excessive collinearity. A detailed summary of retained and excluded features at each preprocessing stage is provided in Table S4 (SI).

All preprocessing and feature selection steps were performed exclusively on the training data within each split to prevent information leakage from the evaluation set.^11^

A split-wise pseudocode description of preprocessing, feature filtering, pairwise construction, and inference is provided in Text S8 to ensure full transparency of the pipeline implementation. Feature scaling was not expected to materially affect the conclusions. While certain models such as logistic regression may benefit from normalization, tree-based models are inherently scale-invariant. Given that the primary focus of this study is on dataset structure and evaluation validity rather than model optimization, scaling was not considered a critical factor.^15,16^

### 2.6 Modeling strategy

Two complementary modeling approaches were considered: a global classification model and a pairwise preference model. The global model estimates the probability that a DES system is optimal within its corresponding (DOI, analyte) group, while the pairwise formulation captures relative preferences between competing systems.

Baseline models, including Logistic Regression, Random Forest, and XGBoost, were evaluated to establish a reference for model performance. Model configurations and hyperparameters are provided in Table S5.

Pairwise preference learning was implemented by constructing feature differences between DES systems within the same group. A hybrid model was defined by combining the global classification probability with aggregated pairwise preference scores, as described in the SI (Text S5). In this study, the pairwise component is not assumed to improve performance *a priori*, but is evaluated as a test of whether limited comparative supervision can provide additional ranking signal beyond the global model.

More complex learning-to-rank algorithms were not used as primary models because the aim was to test the availability of transferable comparative signal under leakage-clean conditions rather than to optimize a high-capacity ranking model on a small and highly redundant pairwise dataset.

The pairwise component is expected to provide complementary but constrained information relative to the global model. In the revised interpretation, the pairwise branch should be viewed primarily as a falsification-oriented probe of whether limited comparative supervision adds ranking value beyond the global model.

### 2.7 Validation and evaluation protocol

A DOI-grouped validation strategy was adopted to approximate realistic generalization across studies, such that all records from the same publication were assigned to the same fold or held-out set. This design prevents study-level information leakage and provides a more reliable estimate of model transferability.^11^

Four complementary evaluation blocks were used in this study: (i) baseline DOI-grouped cross-validation across model classes, (ii) a validation-strategy comparison designed to illustrate performance inflation under random row-wise splitting, (iii) a final leakage-clean DOI-held-out comparison between the baseline full model and the hybrid formulation, and (iv) sanity baseline evaluation using repeated random within-group ranking and a simple leakage-clean heuristic. These analyses serve different purposes and should not be interpreted interchangeably. A schematic summary of these evaluation blocks, including dataset versions, feature spaces, split strategies, and reporting locations, is provided in Table 2.

**Table 2:** Summary of experimental settings used across evaluation blocks

Analysis block	Dataset version	Feature space	Split type	Models	Primary purpose	Reported in
Baseline cross-validation	Clean dataset	Leakage-clean descriptors	DOI-grouped CV	LogReg, RF, XGB	Reference model performance under grouped validation	Tables 3 and S10
Validation strategy comparison	Same dataset	Same feature space	Random *vs.* DOI-grouped	LogReg baseline	Quantify inflation due to improper splitting	Table S11
Final holdout evaluation	Clean dataset	Leakage-clean descriptors	DOI-held-out	LogReg (baseline), hybrid	Assess classification *vs.* recommendation under strict separation	Tables 4 and S16
Restricted comparable-group evaluation	Holdout subset	Same feature space	DOI-held-out (subset)	Baseline *vs.* hybrid	Evaluate ranking under meaningful competition	Tables 4, S7–S9 and S20
Ablation and sensitivity	Holdout subset	Same feature space	DOI-held-out	Full-only, pairwise-only, hybrid	Assess contribution of pairwise component	Tables S12 and S13
Sanity baseline evaluation	Holdout subset	Leakage-clean descriptor subset	DOI-held-out	Random ranking; heuristic baseline	Contextualize group-level ranking against trivial and simple rule-based alternatives	Section 3.5, Tables 5, S17 and Text S10

Model performance was evaluated at both the record and group levels. Record-level classification was assessed using receiver operating characteristic area under the curve (ROC-AUC), precision-recall area under the curve (PR-AUC), Matthews correlation coefficient (MCC), balanced accuracy, precision, recall, and *F*_1_-score.^14^ Group-level recommendation performance was evaluated within each (DOI, analyte) group using Hit@1, Hit@3, and mean reciprocal rank (MRR).

For the hybrid model, ranking was performed using pairwise inference within each group with aggregation of pairwise comparisons, as detailed in the SI. Given the known sparsity of competitive group structures in literature-derived datasets, additional restricted evaluations on comparable multi-candidate groups were performed to assess ranking performance under more meaningful decision contexts.

To contextualize learned model performance against trivial and simple rule-based alternatives, two sanity baselines were additionally evaluated at the group level: repeated random within-group ranking and a deterministic leakage-clean heuristic based on analyte-DES polarity mismatch.

### 2.8 Implementation and reproducibility

All analyses were implemented in Python using RDKit, scikit-learn, XGBoost, pandas, and numpy. The software environment and library versions are reported in Table S14.

The SI provides detailed methodological and computational documentation, including literature search and extraction protocols (Text S1–S3), feature definitions (Text S4), hybrid model formulation (Text S5), and pairwise dataset characterization (Text S6–S7), together with the curated dataset (Dataset S1).

To make train/test isolation explicit, the revised SI now includes split-wise pseudocode for preprocessing, pairwise construction, and within-group ranking inference.

The provided code (Code S1) reproduces the leakage-clean data processing, model training, and evaluation workflows reported in this study. While it ensures reproducibility of the final results, it is not intended as a formal proof of the absence of all possible indirect dependencies beyond the explicitly controlled pipeline steps. The complete reproducibility package, including Dataset S1, Code S1, and the core audit/evaluation outputs, is archived at Zenodo under DOI: https://doi.org/10.5281/zenodo.21257665.

## 3 Results

### 3.1 Dataset structure and target definition

The final leakage-clean modeling dataset comprised 757 experiment-level records from 94 source studies, organized into 626 (DOI, analyte) competitive groups. This final modeling table represents the subset of retained literature sources that contained sufficient experiment-level information, chemical identifiers, recovery values, and descriptor eligibility for inclusion in the leakage-clean pipeline. Each group defines a local comparison space in which DES systems were evaluated for the same analyte within a single study. Despite its apparent size, the dataset exhibited highly uneven informative content. Using a Δrecovery threshold of 5% as an operational diagnostic of meaningful competition, only 21 groups (∼3.4%) were classified as informative, while the remainder were dominated by a single optimum. Qualitative sensitivity to alternative thresholds is shown in Fig. S1.

This structure indicates that the dataset is dominated by coarse discriminative signal—clear separation between optimal and suboptimal systems—while fine-grained ranking signal is scarce. Consequently, the effective sample size for learning relative performance differences is substantially smaller than the nominal number of records. The target variable, is_optimal, was defined within each (DOI, analyte) group based on maximum reported recovery. This definition is inherently contextual and study-specific, reflecting the best-performing system among those tested rather than an absolute chemical optimum. In rare cases, multiple systems shared the optimal label due to ties. Thus, label validity depends on both observed performance and the completeness of the candidate set explored within each study.

**Fig. 2:**
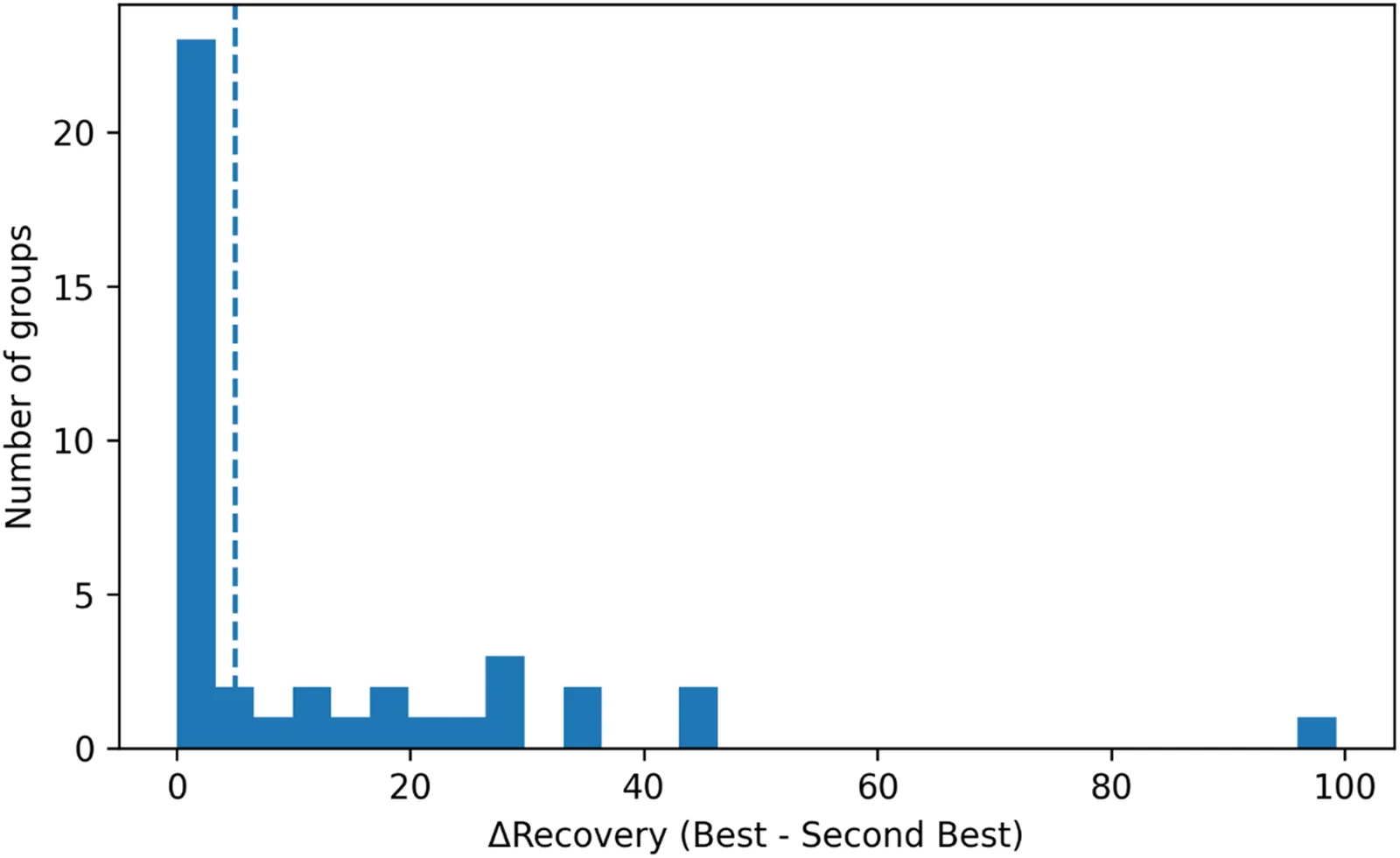
Distribution of recovery gaps between the best and second-best DES systems across (DOI, analyte) groups.

These properties imply that the learning problem is intrinsically local and that limited exploration of near-optimal regions restricts informative comparisons between competing systems. This is further supported by the distribution of recovery gaps within groups (Fig. 2), where most groups show large differences between the best and second-best systems. Taken together, the dataset contains abundant coarse signal but limited information for reliable ranking or recommendation, constraining both statistical power and interpretability of positive labels as transferable optima. The observed class imbalance is an intrinsic consequence of the group-based target definition, where typically a single system is labeled as optimal within each group, and is therefore treated as a structural property rather than a modeling artifact.^17,18^

### 3.2 Baseline modeling under DOI-grouped validation

To establish a realistic reference point, baseline classifiers were evaluated under DOI-grouped cross-validation, in which all records from the same study were assigned to the same fold. This design minimizes study-level information leakage and provides a more stringent estimate of generalization than random row-wise splitting.^11^

Under DOI-grouped validation, all baseline models showed modest and consistent performance (Table 3). ROC-AUC ranged from 0.5657 to 0.5845, while MCC remained near zero (−0.0404 to 0.0238).^19^ Balanced accuracy was also near chance level (0.4845–0.4964), indicating limited discrimination under leakage-controlled conditions.

**Table 3:** Baseline model performance under DOI-grouped cross-validation

Model	No. of features	ROC-AUC (mean ± SD)	PR-AUC (mean ± SD)	MCC (mean ± SD)	Balanced accuracy (mean ± SD)
Logistic regression	75	0.5689 ± 0.1685	0.9263 ± 0.0580	0.0238 ± 0.1098	0.4964 ± 0.0978
XGBoost	75	0.5657 ± 0.1010	0.9263 ± 0.0470	−0.0087 ± 0.0645	0.4845 ± 0.0394
Random forest	75	0.5845 ± 0.1134	0.9347 ± 0.0443	−0.0404 ± 0.0240	0.4913 ± 0.0060

Although PR-AUC values were relatively high, this reflects the prevalence of the positive class under the within-group target construction and should not be interpreted as evidence of decision-level usefulness. MCC and balanced accuracy provide a more conservative assessment of model performance, and fold-level variability (Table S10) confirms that this pattern is consistent across models.

**Fig. 3:**
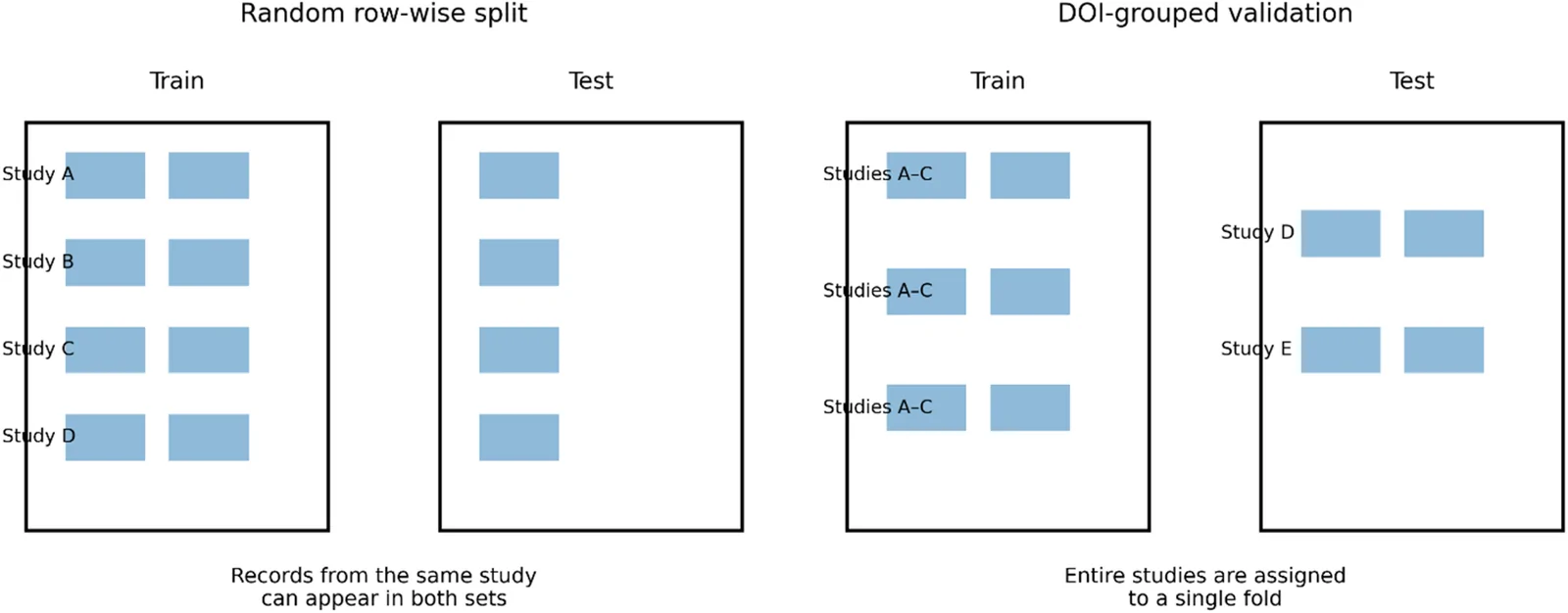
Comparison between random row-wise splitting and DOI-grouped validation.

Comparison with random row-wise splitting highlights the impact of validation design. Performance is strongly inflated under random splitting (Fig. 3 and Table S11), confirming the presence of study-level leakage and reinforcing the need for grouped validation.^11^

Overall, these results indicate that baseline performance is constrained primarily by dataset structure rather than model choice, motivating the leakage-clean reconstruction described below.

### 3.3 Leakage audit and pipeline reconstruction

Inspection of the original pipeline revealed that the pairwise feature space included outcome-derived variables such as recovery, enrichment factor, and LOD, which are not available at prediction time and introduce information leakage (Table 1).

**Fig. 4:**
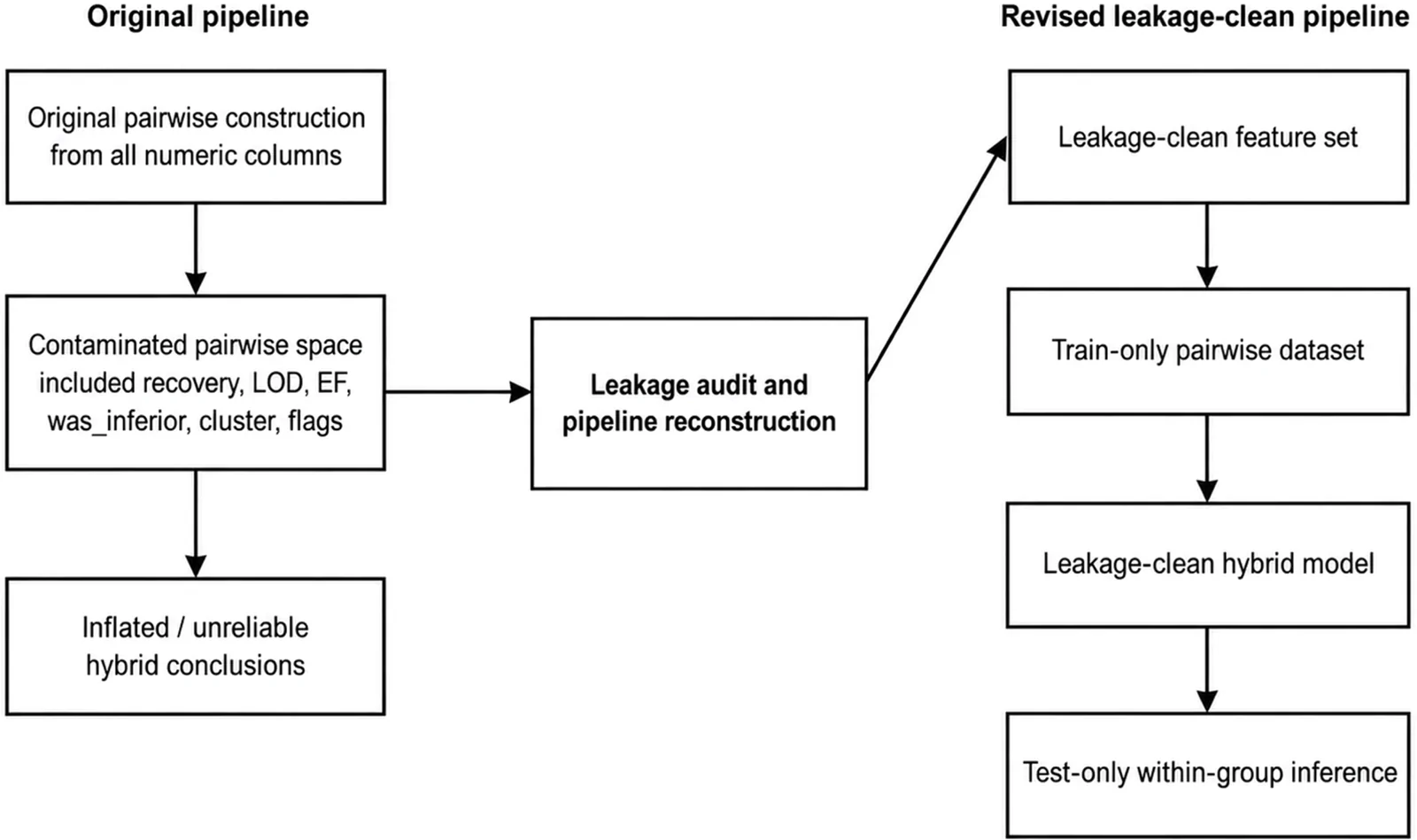
Reconstruction of the hybrid pipeline from contaminated pairwise features to leakage-clean true pairwise inference.

To address this issue, the pipeline was reconstructed using a strictly leakage-clean feature definition. All outcome-related variables were excluded, and only chemically interpretable descriptors were retained (Table S15). The revised workflow (Fig. 4) follows a split-first, training-only paradigm.

Under this corrected framework, previously observed performance gains were not reproduced. Instead, results indicate a more constrained outcome: modest improvement in record-level classification but no stable improvement in recommendation performance under the available comparative supervision. These conclusions remain bounded by the limited pairwise sample size and the restricted descriptor regime.

### 3.4 Leakage-clean holdout evaluation with true pairwise inference

Under DOI-held-out evaluation, the hybrid model showed moderate improvement in record-level classification relative to the baseline (Table S16), indicating improved global discrimination under leakage-controlled conditions.

At the group level, ranking performance was evaluated across both the full held-out set and restricted comparable multi-candidate subsets (Table 4). Full-group metrics were high for both models, but these values are largely descriptive because most held-out groups contained only one candidate or a single dominant optimum. In the comparable subsets, where ranking constitutes a meaningful decision problem, performance decreased substantially. The hybrid model did not outperform the baseline in any non-tied comparable group; point estimates of Hit@1 and MRR were lower for the hybrid model in both ≥2- and ≥3-candidate subsets. Paired effect-size analysis showed negative ΔMRR values for the hybrid model, while exact sign tests remained non-significant because only three non-tied comparable groups were available for formal comparison (Table S20).

**Table 4:** Group-level ranking performance across all held-out groups and restricted comparable multi-candidate subsets^a^

Scope	*n* Groups	Baseline Hit@1	Hybrid Hit@1	Baseline MRR	Hybrid MRR	ΔMRR	Hybrid better	Hybrid worse	Equal	*p*-Value
All groups	119	0.983	0.975	0.987	0.980	−0.0067	0	3	116	0.25
Comparable ≥2	8	0.750	0.625	0.802	0.702	−0.0997	0	3	5	0.25
Comparable ≥3	6	0.667	0.500	0.736	0.603	−0.1329	0	3	3	0.25

a ΔMRR is defined as hybrid minus baseline. Exact sign-test *p*-values were calculated after excluding tied groups. Full-group metrics are reported for completeness but should be interpreted descriptively because most groups contain only one candidate or a single dominant optimum.

These results reflect the structure of the leakage-clean pairwise dataset (Table S6), which is small, highly structured, and derived from a limited number of informative groups. Under these conditions, the available pairwise supervision appears sufficient to capture coarse directional preferences, but not sufficient to support stable additional ranking benefit beyond the baseline model.

### 3.5 Sanity baselines for group-level ranking

To contextualize the practical meaning of the observed ranking performance, two sanity baselines were evaluated under the same leakage-clean DOI-held-out setting: repeated random ranking within each (DOI, analyte) group and a deterministic leakage-clean heuristic based on analyte-DES polarity mismatch.

The random baseline provides a lower-bound reference corresponding to the absence of learned structure. In the full held-out set, random ranking achieved high apparent performance because most groups contain only one candidate or a single dominant optimum. This confirms that full-group ranking metrics can be inflated by trivial group structure and should be interpreted descriptively rather than as direct evidence of recommendation capability.

The heuristic baseline provides a more chemically interpretable reference than random ranking. It ranks candidate DES systems according to increasing analyte-DES polarity mismatch, operationalized as the absolute difference between analyte log *P* and DES-level weighted-mean log *P*. This heuristic was selected because polarity compatibility is a simple, transparent, and leakage-clean physicochemical rule that is directly available from the same descriptor space used by the ML models. It is not intended to represent an optimal DES-selection rule; rather, it serves as a conservative rule-based reference for assessing whether the learned models provide value beyond a simple descriptor-level compatibility principle. Deterministic tie-breaking was performed using TPSA mismatch, hydrogen-bond balance mismatch, and DES system identifier, as detailed in Text S10.

Comparison with these sanity baselines provides two complementary insights (Tables 5 and S17). First, the high performance of random and heuristic baselines in the full held-out set reinforces that full-group ranking metrics are strongly affected by trivial group composition. Second, in comparable multi-candidate groups, the baseline ML model outperformed both random ranking and the polarity-mismatch heuristic in Hit@1 and MRR, indicating that it captures non-trivial structure beyond the simplest descriptor-level rule. However, the hybrid model did not provide stable improvement over the baseline in these same comparable groups. These results support the central interpretation that meaningful recommendation assessment requires restricted comparable-group evaluation rather than reliance on full-group aggregate metrics.

**Table 5:** Comparison of model performance with sanity baselines under leakage-clean DOI-held-out evaluation^a^

Scope	Random Hit@1 (mean ± SD)	Heuristic Hit@1	Baseline Hit@1	Hybrid Hit@1	Random MRR (mean ± SD)	Heuristic MRR	Baseline MRR	Hybrid MRR
All groups	0.951 ± 0.010	0.950	0.983	0.975	0.967 ± 0.006	0.966	0.987	0.980
≥2 candidates	0.273 ± 0.147	0.250	0.750	0.625	0.507 ± 0.095	0.489	0.802	0.702
≥3 candidates	0.193 ± 0.156	0.167	0.667	0.500	0.423 ± 0.113	0.402	0.736	0.603

a Random ranking represents expected performance in the absence of learned structure (mean ± SD). The heuristic baseline is based on analyte-DES polarity mismatch. Full-group metrics are strongly affected by trivial group composition, whereas comparable-group results provide a more meaningful assessment of model ranking behavior.

### 3.6 Descriptor importance and chemical interpretability

To identify the descriptors contributing most strongly to the leakage-clean baseline model, permutation importance analysis was performed on the DOI-held-out test set using ROC-AUC as the scoring metric. The most influential descriptors included analyte hydrogen-bond acceptor count, HBD log *P*, analyte molecular weight, DES-analyte heavy-atom interaction terms, DES hydrogen-bond contrast, DES-level TPSA, analyte log *P*, and hydrogen-bond complementarity features (Table S19).

These descriptors are chemically interpretable and broadly correspond to polarity, molecular size, polar surface area, and hydrogen-bonding compatibility, all of which are expected to influence DES-analyte extraction behavior. The importance profile is also consistent with the sanity-baseline results: the leakage-clean models primarily rely on chemically plausible but coarse compatibility descriptors rather than on fine-grained mechanistic information.

Importantly, the feature-importance pattern should not be interpreted as a causal chemical design rule. The descriptor space remains incomplete and does not explicitly encode several process-level variables, including matrix composition, pH, water content, ionic strength, extraction time, temperature, phase behavior, viscosity, or DES microstructure. Instead, the analysis supports the interpretation that the models primarily learn descriptor-level compatibility patterns within the limits of the available literature-derived feature space.

### 3.7 Group-structure diagnostics and evaluation fragility

**Fig. 5:**
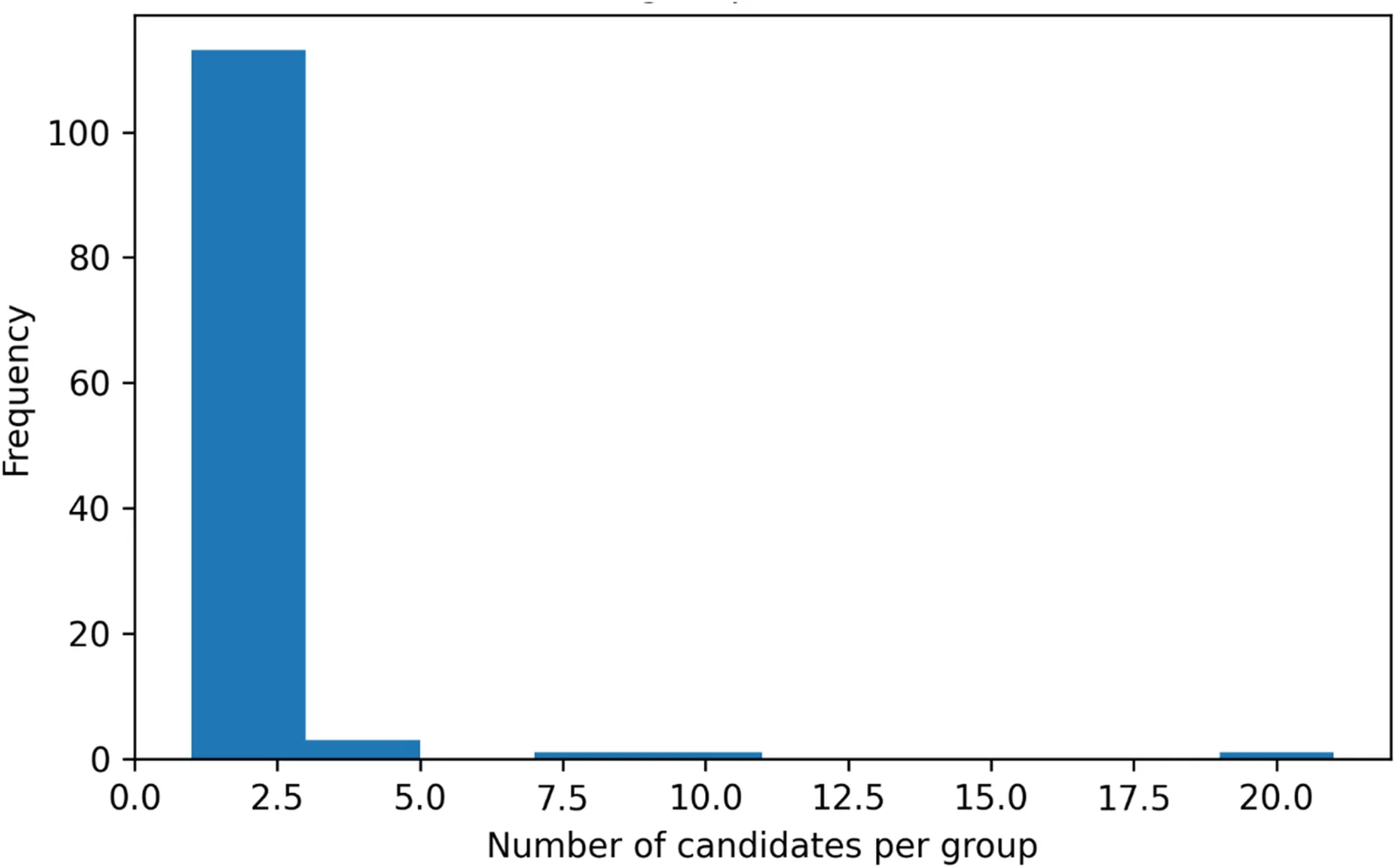
Distribution of group sizes (number of candidates per (DOI, analyte) group).

Analysis of the evaluation space reveals that most (DOI, analyte) groups do not constitute meaningful ranking problems. As shown in Table 6 and Fig. 5, the median group size is one, and the majority of groups contain a single optimal system. This structural sparsity directly affects ranking metrics. Measures such as Hit@1 and MRR assume multiple competing candidates, and when this assumption is violated, metrics become inflated and lose interpretability.^9,11^

**Table 6:** Summary of group structure in the leakage-clean holdout dataset

Metric	Value
*n*_groups	119
mean_group_size	1.386555
median_group_size	1
mean_*n*_positive	1.008403
pct_single_positive_groups	99.15966
pct_tie_for_best_groups	0.840336

This effect is evident when restricting evaluation to comparable multi-candidate groups, where performance decreases substantially (Table S7). Under these conditions, the hybrid model does not outperform the baseline and often performs worse.

Uncertainty analysis further shows that estimates are statistically unstable (Table S8), with wide and overlapping confidence intervals. This indicates that observed differences are not robust. In this study, evaluation fragility is evidenced by the divergence between full-group and restricted evaluation, combined with instability due to the limited number of evaluable groups.

### 3.8 Implications for recommendation validity

Taken together, the leakage-clean holdout evaluation, comparable-group analysis, sanity baselines, and descriptor-importance results indicate that record-level classification gains are not sufficient evidence of reliable DES recommendation.^13,20,21^ The baseline model captures non-trivial descriptor-level structure in comparable groups, but the hybrid formulation does not provide stable additional ranking benefit under the present data regime.

Practically, models trained on literature-derived DES extraction data should therefore be used as diagnostic or prioritization tools rather than autonomous selection tools. Top-ranked DES candidates should be interpreted as experimentally testable hypotheses, particularly when the underlying candidate set lacks comparable near-optimal alternatives.

## 4 Discussion

### 4.1 From model performance to data-structure constraints

The central result of this study is that the achievable performance of literature-derived DES recommendation is constrained primarily by data structure and available supervision. The leakage-clean models retain some ability to discriminate clearly suboptimal systems from locally optimal ones, but the dataset lacks the competitive structure required to learn stable ranking relationships among high-performing alternatives.^19^

This distinction is important because the dataset contains many records but few meaningful decision problems. Most (DOI, analyte) groups contain either a single candidate or a single dominant optimum, so apparent ranking performance can be driven by group composition rather than by genuine candidate selection. Under these conditions, increasing model complexity does not directly address the underlying limitation: the scarcity of diverse, comparable, multi-candidate supervision.^9^

The revised interpretation is therefore data-centric rather than algorithm-centric. The results do not show that ML, pairwise learning, or recommendation modeling are generally ineffective. Instead, they show that under the present literature-derived DES extraction setting, dataset structure and supervision quality define the boundary of what can be inferred.

### 4.2 Metric-utility disconnect in chemical recommendation

The results illustrate a metric-utility disconnect: improved record-level discrimination does not necessarily imply better decision-level recommendation. Classification metrics evaluate separation across individual records, whereas recommendation requires reliable ordering within a local candidate set. These objectives can diverge when the evaluation groups are sparse, imbalanced, or dominated by trivial cases.

In the present dataset, classification gains mainly reflect improved separation between clearly optimal and clearly suboptimal records. They do not provide evidence that the model can reliably distinguish among near-optimal DES candidates within a comparable experimental context. Sanity baselines and restricted comparable-group evaluation are therefore essential, because they reveal whether apparent performance exceeds trivial group structure and simple descriptor-level rules.

This distinction is particularly important for literature-derived chemical datasets, where reported studies often emphasize final optima rather than systematically sampled alternatives. Reliable recommendation evaluation must therefore align the metric, target definition, and group structure with the intended decision problem.^13,22^

### 4.3 Pairwise learning as a diagnostic under sparse comparative supervision

Pairwise preference learning is often considered a natural formulation for recommendation problems. In principle, it is well suited to settings in which multiple candidates are compared within the same decision context and sufficient within-group preference information is available. Therefore, the present analysis should not be interpreted as a general test of pairwise learning as a methodology.

In the leakage-clean reconstruction used here, the available pairwise supervision is extremely limited. The training-only pairwise dataset was constructed from only a small number of informative (DOI, analyte) groups and contains substantial within-group redundancy (Table S6). As a result, the pairwise branch is underpowered for estimating transferable preference relationships across studies and is sensitive to the limited diversity of available comparisons.

For the same reason, more complex learning-to-rank algorithms such as LambdaMART, RankNet, neural rankers, or listwise ranking models were not expected to provide a reliable solution under the present data regime. These methods generally require substantially larger and more diverse query-group or preference datasets than are available here. Applying more flexible ranking algorithms to only a small number of informative training groups would increase model capacity without increasing the underlying comparative information, thereby increasing the risk of overfitting or unstable estimates. The objective of this study was therefore not to benchmark advanced ranking algorithms, but to evaluate whether the literature-derived dataset contains enough leakage-clean comparative supervision to support recommendation claims.

The observed ranking behavior is consistent with this limitation. In comparable-group evaluation, the hybrid model does not show stable improvement over the baseline, and paired effect-size analysis indicates negative ΔMRR point estimates under the current split and target definition (Table 4 and S20). Ablation and sensitivity analyses further show that the pairwise component does not contribute stable additional ranking signal under the present evaluation setting (Tables S12 and S13).^20,21^

Accordingly, the pairwise component should be interpreted primarily as a diagnostic probe rather than as a failed ranking methodology. Its limited benefit indicates that the current literature-derived DES extraction dataset does not provide enough diverse comparative supervision to support a robust pairwise ranking advantage. This conclusion is specific to the present target definition, descriptor regime, and train-only pairwise construction, and should not be generalized to designed datasets, richer experimental comparisons, or other pairwise learning frameworks with substantially larger and more balanced preference data.

### 4.4 Evaluation fragility in literature-derived datasets

The group-structure diagnostics in Section 3.7 reveal a broader issue that we refer to as evaluation fragility. This condition arises when the assumptions of a metric do not match the empirical structure of the dataset. Ranking metrics such as Hit@1 and MRR assume meaningful competition within each group, but most groups in the present holdout set do not satisfy this assumption.

In this study, evaluation fragility is evidenced by three observations: high apparent full-group ranking performance under trivial group structure, substantial degradation under restricted comparable-group evaluation, and wide uncertainty intervals under small evaluable sample sizes. Sanity baselines further show why full-group ranking metrics must be interpreted descriptively rather than as direct evidence of recommendation utility.

Thus, evaluation fragility is not a property of a single model, but of the interaction between data structure, target definition, and metric assumptions. Recognizing this interaction is essential before drawing practical conclusions from ML-based chemical recommendation benchmarks.^11^

### 4.5 Practical implications for DES selection and AI-ready reporting

The findings of this study do not provide a direct rule for selecting a new DES system, nor do they replace experimental validation. Instead, their practical value lies in defining the conditions under which machine-learning-assisted DES recommendation can be interpreted reliably. Under the present literature-derived data structure, ML models may be useful for preliminary prioritization or hypothesis generation, but they should not be treated as autonomous decision tools for selecting optimal DES systems.

For current DES selection workflows, the results suggest that model-generated rankings should be interpreted in relation to the structure of the candidate set. Predictions are more informative when several DES candidates have been tested for the same analyte under comparable experimental conditions. Conversely, when a literature-derived group contains only one candidate or one clearly dominant optimum, high ranking accuracy can be misleading because the evaluation does not represent a meaningful selection problem.

For future experimental studies, the most important implication is that DES screening experiments should be designed to generate decision-relevant comparative data. This requires reporting not only the final optimal DES, but also near-optimal, low-performing, and rejected candidates evaluated under the same experimental context. Such reporting would increase the number of informative multi-candidate groups and improve the ability of ML models to learn ranking relationships among chemically plausible alternatives.

In addition, experimental metadata should be reported in a structured and machine-readable form. At minimum, this should include the full DES composition, HBA/HBD identities, molar ratio, water content when applicable, analyte identity, matrix, pH, extraction time, temperature, ionic strength or salt addition, extraction technique, recovery, uncertainty or replicate information, enrichment factor, and detection-limit units. These variables are essential for distinguishing solvent effects from protocol, matrix, and reporting effects.

Taken together, these recommendations shift the role of ML from retrospective performance maximization toward experimental design diagnostics. A DES dataset suitable for recommendation learning should contain comparable candidate sets, explicit negative and near-optimal examples, standardized metadata, and evaluation units aligned with the intended decision problem. Without these elements, apparent predictive accuracy may reflect reporting structure rather than reliable chemical recommendation capability. These practical recommendations are summarized in Table 7 and expanded as an AI-ready reporting checklist in Text S12 and Table S25.

**Table 7:** Practical implications for AI-ready DES extraction datasets^a^

Practical issue	Recommendation	Expected benefit
DES candidates reported only as final optimum	Report all tested DES systems, including suboptimal and rejected candidates	Preserves within-study competition and enables ranking evaluation
Lack of near-optimal alternatives	Include near-optimal candidates evaluated under the same analyte and protocol	Provides informative comparisons among chemically plausible systems
Sparse or single-candidate groups	Design screening experiments with multiple candidates per analyte	Reduces trivial ranking cases and improves decision-level supervision
Incomplete experimental metadata	Report matrix, pH, water content, ionic strength, extraction time, temperature, and extraction technique	Separates solvent effects from protocol and matrix effects
Ambiguous DES identity	Report HBA/HBD identities, molar ratio, water content, and chemical identifiers when possible	Improves descriptor calculation and reproducibility
Inconsistent performance reporting	Report recovery values with units, replicates, uncertainty, EF, LOD/LOQ units, and provenance	Enables reliable target construction and data auditing
Overreliance on aggregate accuracy	Evaluate models within comparable multi-candidate groups and compare against sanity baselines	Aligns evaluation with practical DES-selection decisions

a The recommendations are intended to improve the design and reporting of DES extraction studies so that future literature-derived datasets contain sufficient comparative structure for reliable ML-based recommendation.

### 4.6 Chemical representation limits of the present leakage-clean feature space

The reporting recommendations above are particularly important because the present feature space is limited by the information consistently available in the literature-derived records. The feature space used in this study is intentionally restricted to leakage-clean, interpretable molecular descriptors. This representation captures broad physicochemical compatibility patterns, including polarity matching, polar surface compatibility, hydrogen-bonding complementarity, approximate molecular size, and DES compositional effects. These descriptors are chemically meaningful and provide a transparent basis for model evaluation; however, they do not fully encode the physicochemical complexity of DES-based extraction.^13^ In particular, extraction performance can depend strongly on matrix effects, pH, water content, ionic strength, extraction time, temperature, phase behavior, viscosity, and DES microstructure. These variables are inconsistently reported across the literature-derived records and therefore could not be included systematically without introducing missingness, bias, or leakage risk. Consequently, the observed performance ceiling reflects both dataset structure and descriptor limitations. Under this representation regime, the model is expected to primarily capture coarse descriptor-level compatibility patterns rather than fine-grained physicochemical mechanisms governing extraction performance.^23,24^

Accordingly, these results should not be interpreted as a general failure of machine learning for DES selection, but as a limitation of applying leakage-clean descriptor-based models to literature-derived datasets with incomplete experimental and physicochemical representation.

### 4.7 Target conditionality and label pathology

The target variable reflects the best observed DES condition within a study-specific candidate set rather than an intrinsic optimum. The positive label therefore depends on which candidates were tested, how completely the study reported them, and under which experimental conditions they were compared.

This conditionality limits the interpretation of predictions. Model outputs should be read as reconstructions of study-bounded relative preference, not as transferable chemical optima.

### 4.8 Alternative target definitions and ranking scope

The target definition used in this study was selected to match the most consistently recoverable decision structure in the literature-derived dataset: identifying the best observed DES system within each study-specific (DOI, analyte) candidate set. However, this is not the only possible formulation of the learning task.

Top-k labeling could assign multiple systems within a group as positive, potentially reducing label sparsity when several candidates perform similarly. However, in the present dataset many groups contain only one candidate or a single clearly dominant optimum, so top-k labeling would not create meaningful additional competition in most groups. In very small groups, top-k labeling may also convert the task into a near-trivial positive-label assignment rather than a ranking problem.

Recovery-threshold labeling provides another possible formulation, for example by labeling all systems within a fixed recovery margin of the best-performing DES as positive. Such a target may be useful in designed datasets where experimental conditions are standardized. In the present literature-derived dataset, however, recovery values are strongly conditioned by matrix composition, pH, water content, extraction time, temperature, ionic strength, and other experimental variables that are inconsistently reported. Applying a universal recovery threshold across heterogeneous studies could therefore introduce a false sense of comparability.

Direct regression on recovery would address a different task: predicting an experimental performance value rather than recommending the best candidate within a local decision context. Regression may be appropriate when recovery values are measured under standardized and well-described conditions, but in this dataset recovery is conditional on study-specific protocols and incomplete metadata. Without systematic information on experimental conditions, regression could conflate solvent effects with matrix, protocol, and reporting effects.

Pairwise Δrecovery-based labels are conceptually aligned with ranking, but they require sufficient within-group comparisons with reliable recovery differences. The present dataset contains too few informative multi-candidate groups to support a robust Δrecovery-based ranking benchmark. Therefore, while alternative targets are scientifically relevant and may be valuable in future designed datasets, they do not remove the central limitation identified here: literature-derived DES extraction data provide sparse and uneven comparative supervision for decision-level recommendation. A structured summary of these alternative target formulations is provided in Text S11 and Table S24.

### 4.9 Study boundaries and limitations

The conclusions of this study are bounded by the present application domain, target definition, descriptor regime, and limited number of comparable groups. They should not be interpreted as a general failure of ML, recommendation modeling, or pairwise learning.

A further limitation is the literature-derived nature of the dataset. Although a random subset of studies was manually validated at the field level and all detected discrepancies were corrected, unvalidated records may still contain residual errors arising from heterogeneous reporting formats, complex optimization figures, dense SI tables, non-standard component nomenclature, visually encoded molar ratios, unit annotations, or incomplete experimental metadata. Such errors are relevant for ranking because small changes in DES identity, analyte assignment, molar ratio, or recovery value can affect descriptor construction, group composition, and optimal-label assignment.

Artificial class balancing techniques such as SMOTE were not applied because they may disrupt the group-based structure of the dataset and introduce synthetic records not grounded in experimental comparisons. In this study, class imbalance is therefore treated as a structural property of the data generation process rather than as a defect to be artificially corrected.^17,25^

### 4.10 Scope of applicability beyond DES extraction

Although the empirical results are specific to literature-derived DES-based pesticide extraction, the diagnostic framework may be applicable to other chemistry-related recommendation problems constructed from heterogeneous literature data. Examples include solvent selection, extraction-condition recommendation, catalyst or material screening, and reaction-condition recommendation, where reported studies often emphasize locally optimal outcomes rather than systematically sampled candidate spaces.

The transferable contribution of this work is therefore methodological rather than algorithm-specific. The combination of leakage auditing, group-aware splitting, train-only preprocessing, restricted comparable-group evaluation, sanity baselines, and uncertainty analysis provides a general framework for assessing whether a literature-derived dataset contains sufficient decision-relevant supervision. However, the numerical findings reported here, including the limited benefit of pairwise augmentation, should not be transferred directly to other domains without re-evaluating the target definition, group structure, descriptor completeness, and availability of informative multi-candidate comparisons.

## 5 Conclusions

This study evaluated whether literature-derived DES extraction data can support machine-learning-based recommendation under leakage-clean and group-aware evaluation. The analysis shows that record-level predictive improvements do not necessarily translate into reliable within-group recommendation.

After leakage-clean reconstruction, the hybrid model improved classification metrics but did not provide stable group-level ranking benefit over the baseline in meaningful comparable groups. Sanity baselines, paired effect-size analysis, and uncertainty estimates indicate that full-group ranking metrics are strongly affected by trivial group structure and should not be interpreted as direct evidence of decision-level utility.

The main limitation is not simply model capacity, but the structure of the available supervision. The dataset contains sufficient signal for coarse discrimination between clearly suboptimal and locally optimal systems, but too few informative multi-candidate groups to support robust ranking among near-optimal alternatives. This limitation is reinforced by the study-bounded target definition, incomplete descriptor representation, and limited train-only pairwise supervision.

Practically, ML models trained on such datasets should be used as diagnostic or prioritization tools rather than autonomous DES-selection systems. Progress in AI-assisted DES recommendation will require better experimental reporting: complete candidate sets, near-optimal and low-performing alternatives, comparable experimental contexts, and structured metadata describing DES composition, analyte identity, matrix, pH, water content, extraction conditions, recovery values, uncertainty, and analytical-performance units.

Overall, the work provides a reproducible, leakage-aware framework for assessing recommendation claims in literature-derived chemical datasets. The conclusions are explicitly bounded to the present DES extraction setting, target definition, descriptor regime, and evaluation framework, and should not be interpreted as a general limitation of ML, pairwise learning, or chemical recommendation systems.

## Author contributions

Hakim Faraji conceived the study, administrated the project, designed the methodology, developed and implemented the computational pipeline, organized and curated the modeling dataset, performed the statistical and machine-learning analyses, validated the analytical outputs, interpreted the results, generated the figures and main findings, and prepared and revised the manuscript. Juan A. Mendez-Perez provided methodological guidance and conceptual input, supervised the computational strategy, and critically reviewed the modeling framework, interpretation of the results, and manuscript. Ruth Rodríguez-Ramos contributed to the initial retrieval of source literature, verified a defined subset of literature-derived records against the corresponding full-text articles and SI files, and reviewed and provided targeted comments on the manuscript text concerning literature collection and data provenance. Bárbara Socas-Rodríguez manually verified extracted experimental records against the original publications, contributed to data quality assessment, and critically reviewed the manuscript. Antonio V. Herrera Herrera supervised aspects of data verification, manually checked extracted experimental records against the original publications, contributed to data quality assessment, and critically reviewed the manuscript. All authors reviewed and approved the final version of the manuscript and agreed to be accountable for their respective contributions.

## Conflicts of interest

The authors declare that they have no known competing financial interests or personal relationships that could have appeared to influence the work reported in this paper.

## Supplementary Material

RA-016-D6RA05453F-s001

## Data Availability

The data underlying this study are available in the published article, the supplementary information (SI), and at Zenodo: https://doi.org/10.5281/zenodo.21257665. The repository includes the curated experiment-level dataset (Dataset S1), the reproducible computational workflow (Code S1), and the core audit, evaluation, and reproducibility outputs required to reproduce the analyses reported in this work. The computational workflow and code used to process the data, train the models, perform leakage-clean evaluation, and generate the reported results are available in the same Zenodo repository. All files are publicly accessible without restriction. Supplementary information is available. See DOI: https://doi.org/10.1039/d6ra05453f.
