# Supplementary material for: When high accuracy misleads in literature-derived machine learning for deep eutectic solvent recommendation
Source: RSC Adv. 2026 Aug 4;16(40):45929–42. doi: 10.1039/d6ra05453f (PMC13434746; doi:10.1039/d6ra05453f)
Supplement: Supplementary file 1 [file RA-016-D6RA05453F-s001.pdf]

Supplementary Information (SI) for RSC Advances.
This journal is © The Royal Society of Chemistry 2026

Supporting Information

When High Accuracy Misleads in Literature-Derived Machine Learning for Deep Eutectic Solvent Recommendation

Hakim Faraji [a], Juan A. Mendez-Perez [b], Ruth Rodríguez-Ramos [a, c], Bárbara Socas-Rodríguez [a, d], Antonio V. Herrera Herrera [a, d]

[a] Departamento de Química, Área de Química Analítica, Facultad de Ciencias, Universidad de La Laguna (ULL), Tenerife, Spain

[b] Departamento de Ingeniería Informática y de Sistemas, Universidad de La Laguna (ULL), Tenerife, Spain

[c] CIMO, LA SusTEC, Instituto Politécnico de Bragança, Campus de Santa Apolónia, Bragança, 5300-253, Portugal

[d] Instituto Universitario de Bio-Orgánica Antonio González, Universidad de La Laguna (ULL), Tenerife, Spain

Structure of this Supporting Information

This Supporting Information is organized into five sections:

1. Literature search and data extraction
2. Dataset construction and feature engineering
3. Modeling framework
4. Evaluation and robustness analyses
5. Reproducibility resources

Section S1 — Literature Search and Data Extraction

Text S1. Search Strategy and Study Selection

A structured literature search was conducted in Scopus to identify studies reporting the application of deep eutectic solvents (DESs) in pesticide extraction. The search covered publications available up to 30 December 2025 and was performed using the following query:

TITLE-ABS-KEY (("deep eutectic solvent*" OR "DES" OR "natural deep eutectic solvent*" OR "NADES") AND (pesticide* OR insecticide* OR herbicide* OR fungicide* OR agrochemical*) AND (extraction OR "sample preparation" OR microextraction))

The Scopus search identified 207 research articles. Full texts were obtained for 197 articles. These articles were screened for relevance to DES-based pesticide determination or extraction. Articles that contained DES- and pesticide-related keywords but did not report DES-based pesticide measurement, extraction performance, or experiment-level extraction data were excluded. Following full-text screening, 132 studies were retained for experiment-level data extraction.

Study selection was conducted in two stages. First, titles and abstracts were screened to identify potentially relevant studies. Second, full-text articles were evaluated for eligibility based on predefined inclusion and exclusion criteria.

Studies were included if:

- DES systems were used as extraction solvents in analytical workflows;
- extraction performance metrics (e.g., recovery) were reported at the experiment level;
- sufficient information was available to assign performance values to individual DES systems.

Studies were excluded if:

- DESs were used only as components of materials (e.g., sorbents or composites);
- extraction performance could not be attributed to individual DES systems;
- the study focused solely on physicochemical property prediction;
- experimental details were insufficient to reconstruct experiment-level records.
- Following full-text screening, 132 studies were retained for experiment-level data extraction.

Text S2. AI-Assisted Experiment-Level Data Extraction and Validation

Extraction protocol

Experiment-level data were extracted using a structured prompt-based workflow implemented in NotebookLM, designed to capture all reported DES systems without aggregation or inference. Each record corresponded to a unique combination of DES composition (HBA, HBD, molar ratio), analyte identity, and reported performance metrics.

A strict zero-inference policy was applied: only explicitly reported values were recorded, and missing values were retained as “N/A”. Both optimal and suboptimal systems were included to preserve within-study comparability.

Manual validation and extraction audit

To assess the reliability of the AI-assisted extraction workflow, a random subset of 40 studies was selected from the 132 studies retained for experiment-level extraction, corresponding to approximately 30% of the retained extraction studies. Validation was performed manually against the original publications and their Supporting Information files.

The validation was conducted primarily at the field level, because individual extraction records contain multiple fields that can independently affect DES identity, analyte identity, grouping, target assignment, descriptor construction, or interpretation of experimental context. The core audited fields were HBA identity, HBD identity, molar ratio, analyte identity, and recovery (%). When inconsistencies were observed, related metadata fields such as extraction time, temperature, LOD units, and provenance were also checked against the original source.

A total of 1200 fields were manually checked across the validation subset. Of these, 46 discrepancies were identified prior to correction, corresponding to a pre-correction field-level extraction accuracy of 96.2%. All identified discrepancies were corrected by direct comparison with the source publications and their Supporting Information files before construction of the final modeling table.

Error characterization

The discrepancies were not uniformly distributed across studies or fields. Most discrepancies arose from heterogeneous reporting formats, complex optimization figures, dense tabulated Supporting Information, non-standard component names, or ambiguous visual encoding of experimental conditions. The most common error categories included component-name normalization, molar-ratio parsing, analyte-specific recovery assignment, unit interpretation, extraction-condition assignment, and provenance tracking. Representative corrected entries are provided in Table S22.

These discrepancies were resolved manually by checking the corresponding tables, figures, optimization sections, footnotes, and Supporting Information files of the original publications. When a value could not be explicitly verified, it was not inferred and was retained as missing or excluded according to downstream eligibility rules.

Interpretation of validation results

The reported accuracy reflects field-level agreement prior to correction and should be interpreted as an audit of extraction consistency under the applied protocol. After correction, all validated entries were aligned with the source publications.

Because the full dataset is literature-derived and reporting formats vary substantially across studies, this validation cannot guarantee that all unvalidated records are free of residual errors. Potential remaining errors are most likely to arise from the same categories observed during validation: complex graphical encoding, non-standard component naming, fractional molar ratios, multi-analyte tables, unit annotations in footnotes, and incomplete reporting of experimental conditions. For this reason, the dataset should be interpreted as a curated and audited literature-derived modeling table rather than as an error-free reconstruction of all original experimental contexts.

A quantitative summary of the field-level validation audit, including error counts and accuracy estimates, is provided in Table S1.

Table S1. Summary of extraction validation (field-level audit)

Metric	Value
Scopus records identified	207 research articles
Full-text PDFs and Supporting Information files obtained	197 articles
Studies retained for experiment-level extraction	132 studies
Studies represented in final modeling table	94 studies
Manual validation subset	40 studies
Validation coverage	40/132 retained extraction studies (≈30%)
Validation approach	Random study-level sampling followed by field-level audit
Validation unit	Individual extracted fields
Core fields evaluated	HBA identity; HBD identity; molar ratio; analyte identity; recovery (%)
Additional fields checked when inconsistencies were observed	Extraction time; temperature; LOD units; provenance
Total fields evaluated	1200
Fields with discrepancies before correction	46
Field-level extraction accuracy before correction	96.2%
Correction method	Manual verification against original publications and Supporting Information files
Post-correction status	All identified discrepancies in the validation subset corrected
Most common discrepancy sources	Figure/table parsing; name normalization; molar-ratio parsing; analyte assignment; recovery assignment; unit interpretation
Higher-complexity cases	Visually encoded ratios; dense SI tables; fractional ratios; multi-analyte tables; multi-component DES systems
Representative corrected entries	Table S22
Remaining limitation	Unvalidated records may retain residual systematic errors associated with heterogeneous reporting formats

Section S2 — Dataset and Feature Engineering

Text S3. Notebook LM Extraction Prompt

You are an elite, zero-hallucination AI Scientific Copilot specialized in Deep Eutectic Solvents (DES), Green Analytical Chemistry, and Experiment-Level Data Engineering.
Your mission is to extract a COMPLETE experiment-level dataset from DES-based pesticide/toxin extraction papers.
CRITICAL UPGRADE FROM v5.1:
You MUST extract ALL tested DES systems — not only the optimal one.
Do NOT collapse results to best-performing DES.
Extract sub-optimal, low-performing, and failed systems if reported.
I. NON-NEGOTIABLE EXTRACTION RULES
1) Extract ONLY explicitly reported experimental data.
2) NEVER infer missing values.
3) If a value is not explicitly stated → return "N/A".
4) If multiple DES systems are evaluated in tables or optimization sections:
 - Extract EACH row as a separate experiment entry.
 - Do NOT summarize.
 - Do NOT select only optimal conditions.
5) If a DES is reported as inferior or rejected, still extract it.
6) If recovery is reported for multiple analytes, extract each analyte as a separate entry.
II. PRIORITY TARGET
Focus ONLY on:
- DES used as extraction solvent
- Optimization tables comparing different DES systems
- Effect-of-DES-type studies
- Screening tables
If the paper is purely adsorption material (DES-modified composite) and does NOT report extraction recovery:
Return:

```
{
  "Article_Type": "Non-Extraction (Material Study)",
  "Reason": "No solvent extraction recovery data reported"
}
```

and STOP.
III. OUTPUT STRUCTURE (MANDATORY JSON FORMAT)

```
{
  "Bibliographic_Metadata": {
    "Article_Title": "",
    "Journal_Name": "",
    "DOI": "",
    "Publication_Year": "",
    "Extraction_Confidence_Score": 0-100
  },
  "Experiment_Level_Data": [
    {
      "DES_HBA_Name": "",
      "DES_HBD_Name": "",
      "Molar_Ratio": "",
      "Water_Content_Wt_Percent": float or "N/A",
      "Analyte_Name": "",
      "Analyte_Family": "",
      "Matrix_Category": "Water / Food / Soil / Biological / N/A",
```

```
    "Extraction_Technique": "",
    "Sample_pH": float or "N/A",
    "Extraction_Time_min": float or "N/A",
    "Temperature_C": float or "N/A",
    "Recovery_Percent": float or "N/A",
    "Enrichment_Factor": float or "N/A",
    "LOD_ug_per_L": float or "N/A",
    "Is_Optimal_System": true/false,
    "Was_Reported_As_Inferior": true/false,
    "Provenance": "Exact table/figure/section reference",
    "Confidence_Score": 0-100
   }
  ]
}
```

IV. SCREENING ENFORCEMENT LOGIC
For any table titled similar to:
- “Effect of DES type”
- “Optimization of DES”
- “Comparison of different DES”
- “Screening of DES systems”
You MUST:
- Extract ALL rows.
- Each DES system = one object inside Experiment_Level_Data.
- If recovery is lower than optimal → mark:
  "Was_Reported_As_Inferior": true
- If text explicitly says “optimal DES” → mark:
  "Is_Optimal_System": true
If not specified → set both to false.
V. IMPORTANT
DO NOT:
- Collapse multiple DES into one.
- Ignore low-recovery systems.
- Replace table values with text summary.
- Merge multiple analytes into one record.
If no screening table exists and only one DES tested:
Return single entry in Experiment_Level_Data.
VI. FINAL CHECK BEFORE OUTPUT
Before finalizing JSON:
Count how many DES systems were tested.
If more than one DES is mentioned in the paper and you output only one → YOU FAILED.
END OF PROTOCOL

Text S4. Interaction Feature Definitions

To represent relationships between DES systems and analytes, a set of engineered interaction features was constructed from interpretable molecular descriptors. These features were designed to capture both the internal composition of the DES and its physicochemical relationship to the analyte.

Chemical rationale of descriptor families

The descriptor families were selected to provide a leakage-clean and interpretable approximation of DES–analyte compatibility. LogP-related descriptors were used as coarse proxies for hydrophobicity and polarity matching, which are relevant to analyte partitioning into the DES-rich phase. TPSA-related descriptors represent polar surface compatibility, while hydrogen-bond donor and acceptor descriptors capture possible donor–acceptor complementarity between analytes and DES components. Molecular weight and heavy-atom descriptors provide approximate size and dispersion-related information, whereas ring count and rotatable-bond descriptors provide simple structural and flexibility proxies. Molar-ratio-weighted descriptors were used to preserve composition-dependent information of the DES system. DES–analyte absolute differences were interpreted as mismatch descriptors, ratios as relative proportional contrasts, and products as simple interaction-strength proxies.

These features should be interpreted as low-dimensional physicochemical proxies rather than complete mechanistic descriptors. They do not explicitly represent matrix effects, pH, water content, ionic strength, viscosity, phase behavior, temperature, extraction time, or DES microstructure, because such information was not consistently available across the literature-derived records.

First, DES-level aggregate descriptors were computed from HBA and HBD component descriptors using a molar-ratio-weighted mean:

$$x^{wmean}_{DES} = \frac{n_{HBA}\, x_{HBA} + n_{HBD}\, x_{HBD}}{n_{HBA} + n_{HBD}}$$

where n_{HBA} and n_{HBD} are the parsed molar ratio coefficients of the hydrogen bond acceptor (HBA) and hydrogen bond donor (HBD), respectively.

In addition, within-DES contrast descriptors were defined as absolute differences between HBA and HBD component values:

$$x^{absdiff}_{DES} = |x_{HBA} - x_{HBD}|$$

To characterize DES–analyte relationships, three generic transformations were applied for each descriptor family (molecular weight, logP, TPSA, hydrogen-bond acceptor count, hydrogen-bond donor count, rotatable bonds, ring count, and heavy atom count):

Absolute difference:

$$|x_{analyte} - x^{wmean}_{DES}|$$

Ratio:

$$\frac{x_{analyte}}{x^{wmean}_{DES}}$$

Product:

$$x_{analyte} \cdot x^{wmean}_{DES}$$

These transformations provide complementary views of compatibility. Absolute differences capture mismatch, ratios provide scale-sensitive comparisons, and products provide simple interaction-strength proxies.

Additional physically motivated proxy features were defined to approximate polarity and surface compatibility. A binary polarity-match feature was introduced as:

$$I\left(|\log P_{analyte} - \log P^{wmean}_{DES}| \leq 1.5\right)$$

Similarly, a TPSA-match feature was defined as:

$$I\left(|TPSA_{analyte} - TPSA^{wmean}_{DES}| \leq 20.0\right)$$

Hydrogen-bond complementarity was approximated using the following interaction terms:

$$HBA_{analyte} \cdot HBD^{wmean}_{DES}$$

$$HBD_{analyte} \cdot HBA^{wmean}_{DES}$$

and their combined form:

$$\left(HBA_{analyte} \cdot HBD^{wmean}_{DES}\right) + \left(HBD_{analyte} \cdot HBA^{wmean}_{DES}\right)$$

A hydrogen-bond balance mismatch feature was defined as:

$$|HBA_{analyte} - HBD^{wmean}_{DES}| + |HBD_{analyte} - HBA^{wmean}_{DES}|$$

Finally, molar-ratio-derived descriptors were included to preserve compositional information:

HBA fraction:

$$\frac{n_{HBA}}{n_{HBA} + n_{HBD}}$$

HBD fraction:

$$\frac{n_{HBD}}{n_{HBA} + n_{HBD}}$$

HBA-to-HBD ratio:

$$\frac{n_{HBA}}{n_{HBD}}$$

Together, these features provide a low-dimensional and interpretable representation of DES composition, internal contrast, and DES–analyte compatibility under leakage-clean conditions.

Table S2. Mathematical definitions of engineered features used to represent DES systems and DES–analyte interactions

**Feature Type**	**Formula**	**Description**
Weighted mean	$x^{wmean}_{DES} = \frac{n_{HBA}x_{HBA} + n_{HBD}x_{HBD}}{n_{HBA} + n_{HBD}}$	Composition-weighted descriptor reflecting DES composition, where n_{HBA} and n_{HBD} are the parsed molar ratio parts.
Absolute difference	$\vert x_1 - x_2\vert$	Magnitude of difference between two descriptor values
Ratio	$\frac{x_A}{x_B}$	Scale-sensitive proportional relationship between two descriptor values. Computed only when the denominator is non-zero.
Product	$x_A \times x_B$	Simple interaction-strength proxy based on simultaneous contribution of two descriptor values.
DES aggregate contrast	$\vert x_{\mathrm{HBA}} - x_{\mathrm{HBD}}\vert$	Absolute difference between descriptor values of HBA and HBD components, representing internal compositional heterogeneity within the DES

		system.
DES–analyte absolute difference (Δ descriptor)	$\Delta x = x_{DES} - x_{analyte}$	Relative difference between DES and analyte properties
DES–analyte ratio	$\frac{x_{Analyte}}{x_{DES}^{wmean}}$	Relative proportional contrast between analyte and DES-level descriptor.
DES–analyte product	$x_{Analyte} \times x_{DES}^{wmean}$	Product-based interaction proxy between analyte and DES-level descriptor.
H-bond acceptor–donor product	$HBA_{Analyte} \times HBD_{DES}^{wmean}$	Proxy for complementarity between analyte hydrogen-bond acceptor capacity and DES donor capacity.
H-bond donor–acceptor product	$HBD_{Analyte} \times HBA_{DES}^{wmean}$	Proxy for complementarity between analyte hydrogen-bond donor capacity and DES acceptor capacity.
H-bond complement sum	$(HBA_{Analyte} \times HBD_{DES}^{wmean}) + (HBD_{Analyte} \times HBA^{w}$	Aggregate proxy for total donor–acceptor complementarity.
H-bond balance	$HBD_{DES} - HBA_{analyte}$	Hydrogen-bond complementarity proxy
Polarity mismatch (binary)	$\lvert \log P_{DES} - \log P_{analyte} \rvert$	Absolute difference in polarity (like-dissolves-like principle)
TPSA match (binary)	$1(\lvert TPSA_{Analyte} - TPSA_{DES}^{wmean} \rvert \leq 20.0)$	Binary indicator of approximate polar surface compatibility between analyte and DES, defined using a fixed threshold on the difference in topological polar surface area (TPSA).
Ratio fraction (HBA)	$\frac{n_{HBA}}{n_{HBA} + n_{HBD}}$	Fractional contribution of HBA in the parsed binary DES molar ratio.
Ratio fraction (HBD)	$\frac{n_{HBD}}{n_{HBA} + n_{HBD}}$	Fractional contribution of HBD in the parsed binary DES molar ratio.
HBA-to-HBD ratio	$\frac{n_{HBA}}{n_{HBD}}$	Direct molar ratio between HBA and HBD parts.

Table S3. Molecular descriptors and derived features used for model development. Descriptors were computed from canonical SMILES representations using RDKit and include component-level descriptors, DES-level aggregates, and DES–analyte interaction features.

Feature	Description
analyte_hba	Hydrogen bond acceptor count of the analyte
analyte_hbd	Hydrogen bond donor count of the analyte
analyte_heavyatoms	Number of heavy atoms of the analyte
analyte_logp	Octanol–water partition coefficient (logP) of the analyte
analyte_molwt	Molecular weight of the analyte
analyte_ringcount	Number of rings of the analyte
analyte_rotbonds	Number of rotatable bonds of the analyte
analyte_tpsa	Topological polar surface area (TPSA) of the analyte
des_hba_absdiff	Absolute difference in hydrogen bond acceptor count between HBA and HBD components (DES internal contrast)
des_hba_wmean	Molar-ratio-weighted mean of hydrogen bond acceptor count for the DES
des_hbd_absdiff	Absolute difference in hydrogen bond donor count between HBA and HBD components
des_hbd_wmean	Molar-ratio-weighted mean of hydrogen bond donor count for the DES
des_logp_absdiff	Absolute difference in logP between HBA and HBD components
des_logp_wmean	Molar-ratio-weighted mean of logP for the DES
des_molwt_absdiff	Absolute difference in molecular weight between HBA and HBD components
des_molwt_wmean	Molar-ratio-weighted mean of molecular weight for the DES

des_ringcount_absdiff	Absolute difference in ring count between HBA and HBD components
des_ringcount_wmean	Molar-ratio-weighted mean of ring count for the DES
des_rotbonds_absdiff	Absolute difference in number of rotatable bonds between HBA and HBD components
des_rotbonds_wmean	Molar-ratio-weighted mean of number of rotatable bonds for the DES
des_tpsa_absdiff	Absolute difference in TPSA between HBA and HBD components
des_tpsa_wmean	Molar-ratio-weighted mean of TPSA for the DES
hba_hba	Hydrogen bond acceptor count of the HBA component
hba_hbd	Hydrogen bond donor count of the HBA component
hba_logp	LogP of the HBA component
hba_molwt	Molecular weight of the HBA component
hba_ringcount	Number of rings of the HBA component
hba_rotbonds	Number of rotatable bonds of the HBA component
hba_tpsa	TPSA of the HBA component
hbd_hba	Hydrogen bond acceptor counts of the HBD component
hbd_hbd	Hydrogen bond donor count of the HBD component
hbd_logp	LogP of the HBD component
hbd_molwt	Molecular weight of the HBD component
hbd_ringcount	Number of rings of the HBD component
hbd_rotbonds	Number of rotatable bonds of the HBD component
hbd_tpsa	TPSA of the HBD component
pair_hba_absdiff	Absolute difference between analyte and DES-level hydrogen bond acceptor count
pair_hba_product	Product of analyte and DES-level hydrogen bond acceptor count
pair_hba_ratio	Ratio between analyte and DES-level hydrogen bond acceptor count
pair_hbd_absdiff	Absolute difference between analyte and DES-level hydrogen bond donor count
pair_hbd_product	Product of analyte and DES-level hydrogen bond donor count
pair_hbd_ratio	Ratio between analyte and DES-level hydrogen bond donor count
pair_hbond_acceptor_donor_product	Product of analyte HBA and DES donor capacity (HBD)
pair_hbond_donor_acceptor_product	Product of analyte HBD and DES acceptor capacity (HBA)
pair_hbond_complement_sum	Combined hydrogen-bond complementarity between analyte and DES
pair_hbond_balance_absdiff	Hydrogen-bond balance mismatch between analyte and DES
pair_heavyatoms_absdiff	Absolute difference between analyte and DES-level heavy atom count
pair_heavyatoms_product	Product of analyte and DES-level heavy atom count
pair_heavyatoms_ratio	Ratio between analyte and DES-level heavy atom count
pair_logp_absdiff	Absolute difference between analyte and DES-level logP
pair_logp_match_binary	Binary indicator of approximate polarity match between analyte and DES
pair_logp_product	Product of analyte and DES-level logP
pair_logp_ratio	Ratio between analyte and DES-level logP
pair_molwt_absdiff	Absolute difference between analyte and DES-level molecular weight
pair_molwt_product	Product of analyte and DES-level molecular weight
pair_molwt_ratio	Ratio between analyte and DES-level molecular weight
pair_ringcount_absdiff	Absolute difference between analyte and DES-level ring count
pair_ringcount_product	Product of analyte and DES-level ring count
pair_rotbonds_absdiff	Absolute difference between analyte and DES-level rotatable bonds
pair_rotbonds_product	Product of analyte and DES-level rotatable bonds
pair_rotbonds_ratio	Ratio between analyte and DES-level rotatable bonds
pair_tpsa_absdiff	Absolute difference between analyte and DES-level TPSA
pair_tpsa_match_binary	Binary indicator of approximate TPSA match between analyte and DES
pair_tpsa_product	Product of analyte and DES-level TPSA
pair_tpsa_ratio	Ratio between analyte and DES-level TPSA
ratio_hba_fraction	Fractional contribution of HBA in the DES molar ratio
ratio_hba_part	Parsed HBA coefficient in the DES molar ratio
ratio_hba_to_hbd	HBA-to-HBD molar ratio

ratio_hbd_part	Parsed HBD coefficient in the DES molar ratio

Table S4. Features excluded during preprocessing and the corresponding reasons for removal, including leakage risk, low variance, or missing values.

Feature name	Missing fraction train	Drop reason stage	Selected for stage5
ratio_hba_part	0	keep_after_missingness	TRUE
ratio_hbd_part	0	keep_after_missingness	TRUE
ratio_hba_fraction	0	keep_after_missingness	TRUE
ratio_hbd_fraction	0	drop_high_correlation	FALSE
ratio_hba_to_hbd	0	keep_after_missingness	TRUE
analyte_hba	0	keep_after_missingness	TRUE
analyte_hbd	0	keep_after_missingness	TRUE
analyte_heavyatoms	0	keep_after_missingness	TRUE
analyte_logp	0	keep_after_missingness	TRUE
analyte_molwt	0	keep_after_missingness	TRUE
analyte_ringcount	0	keep_after_missingness	TRUE
analyte_rotbonds	0	keep_after_missingness	TRUE
analyte_tpsa	0	keep_after_missingness	TRUE
des_hba_absdiff	0	keep_after_missingness	TRUE
des_hba_wmean	0	keep_after_missingness	TRUE
des_hbd_absdiff	0	keep_after_missingness	TRUE
des_hbd_wmean	0	keep_after_missingness	TRUE
des_heavyatoms_absdiff	0	drop_high_correlation	FALSE
des_heavyatoms_wmean	0	drop_high_correlation	FALSE
des_logp_absdiff	0	keep_after_missingness	TRUE
des_logp_wmean	0	keep_after_missingness	TRUE
des_molwt_absdiff	0	keep_after_missingness	TRUE
des_molwt_wmean	0	keep_after_missingness	TRUE
des_ringcount_absdiff	0	keep_after_missingness	TRUE
des_ringcount_wmean	0	keep_after_missingness	TRUE
des_rotbonds_absdiff	0	keep_after_missingness	TRUE
des_rotbonds_wmean	0	keep_after_missingness	TRUE
des_tpsa_absdiff	0	keep_after_missingness	TRUE
des_tpsa_wmean	0	keep_after_missingness	TRUE
hba_descriptor_eligible_phase1	0	drop_constant	FALSE
hba_hba	0	keep_after_missingness	TRUE
hba_hbd	0	keep_after_missingness	TRUE
hba_heavyatoms	0	drop_high_correlation	FALSE
hba_logp	0	keep_after_missingness	TRUE

hba_molwt	0	keep_after_missingness	TRUE
hba_ringcount	0	keep_after_missingness	TRUE
hba_rotbonds	0	keep_after_missingness	TRUE
hba_tpsa	0	keep_after_missingness	TRUE
hbd_descriptor_eligible_phase1	0	drop_constant	FALSE
hbd_hba	0	keep_after_missingness	TRUE
hbd_hbd	0	keep_after_missingness	TRUE
hbd_heavyatoms	0	drop_high_correlation	FALSE
hbd_logp	0	keep_after_missingness	TRUE
hbd_molwt	0	keep_after_missingness	TRUE
hbd_ringcount	0	keep_after_missingness	TRUE
hbd_rotbonds	0	keep_after_missingness	TRUE
hbd_tpsa	0	keep_after_missingness	TRUE
pair_hba_absdiff	0	keep_after_missingness	TRUE
pair_hba_product	0	keep_after_missingness	TRUE
pair_hba_ratio	0	keep_after_missingness	TRUE
pair_hbd_absdiff	0	keep_after_missingness	TRUE
pair_hbd_product	0	keep_after_missingness	TRUE
pair_hbd_ratio	0	keep_after_missingness	TRUE
pair_hbond_acceptor_donor_product	0	keep_after_missingness	TRUE
pair_hbond_balance_absdiff	0	keep_after_missingness	TRUE
pair_hbond_complement_sum	0	keep_after_missingness	TRUE
pair_hbond_donor_acceptor_product	0	keep_after_missingness	TRUE
pair_heavyatoms_absdiff	0	keep_after_missingness	TRUE
pair_heavyatoms_product	0	keep_after_missingness	TRUE
pair_heavyatoms_ratio	0	keep_after_missingness	TRUE
pair_logp_absdiff	0	keep_after_missingness	TRUE
pair_logp_match_binary	0	keep_after_missingness	TRUE
pair_logp_product	0	keep_after_missingness	TRUE
pair_logp_ratio	0	keep_after_missingness	TRUE
pair_molwt_absdiff	0	keep_after_missingness	TRUE
pair_molwt_product	0	keep_after_missingness	TRUE
pair_molwt_ratio	0	keep_after_missingness	TRUE
pair_ringcount_absdiff	0	keep_after_missingness	TRUE
pair_ringcount_product	0	keep_after_missingness	TRUE
pair_ringcount_ratio	0.326014	drop_high_missingness	FALSE
pair_rotbonds_absdiff	0	keep_after_missingness	TRUE
pair_rotbonds_product	0	keep_after_missingness	TRUE
pair_rotbonds_ratio	0	keep_after_missingness	TRUE
pair_tpsa_absdiff	0	keep_after_missingness	TRUE
pair_tpsa_match_binary	0	keep_after_missingness	TRUE
pair_tpsa_product	0	keep_after_missingness	TRUE
pair_tpsa_ratio	0	keep_after_missingness	TRUE
ratio_parse_ok	0	drop_constant	FALSE

Section S3 — Modeling Framework

Text S5. Mathematical Formulation of the Hybrid Model

The hybrid model combines a global classification component with a pairwise preference component to produce a ranking of candidate DES systems within each (DOI, analyte) group.

Let x_i denote the feature vector corresponding to DES–analyte record i. The global classification model estimates the probability that a given DES system is optimal within its competitive group:

$$P(y_i = 1|x_i)$$

where $y_i = 1$ indicates that the DES system is labeled as optimal within its (DOI, analyte) group.

To model relative preferences between candidate systems, a pairwise formulation is introduced. For a pair of DES systems (i,j) within the same group, the pairwise feature vector is defined as:

$$\Delta x_{ij} = x_i - x_j$$

The pairwise model produces a directional preference score:

$$f(\Delta x_{ij})$$

which is converted to a probability using a sigmoid transformation:

$$p_{ij} = \frac{1}{1 + exp(-f(\Delta x_{ij}))}$$

where p_{ij} represents the probability that system i is preferred over system j.

For each (DOI, analyte) group containing n candidate DES systems, pairwise comparisons are performed between all pairs, and the pairwise scores are aggregated to obtain a group-level preference score:

$$S_i^{pair} = \frac{1}{n-1}\sum_{j \neq i} p_{ij}$$

The final hybrid score is defined as a weighted combination of the global classification probability and the aggregated pairwise score:

$$S_i^{final} = \alpha_{full} \cdot P(y_i = 1|x_i) + \alpha_{pair} \cdot S_i^{pair}$$

subject to:

$$\alpha_{full} + \alpha_{pair} = 1$$

In this study, fixed weights of $\alpha_{full} = 0.7$ and $\alpha_{pair} = 0.3$ were used to prioritize the stability of the global classification component while incorporating complementary comparative information.

Importantly, all components of the hybrid model are constructed using leakage-clean features, and pairwise inference is performed exclusively within each evaluation group. No outcome-derived variables are used in either component.

Given the limited number of informative (DOI, analyte) groups and the high redundancy of pairwise comparisons (Table S6), the pairwise component should be interpreted as providing complementary but structurally constrained information. Under these conditions, the hybrid formulation should be interpreted as an exploratory diagnostic of whether limited comparative supervision provides additional ranking signal, rather than as a definitive test of pairwise learning in general.

Advanced learning-to-rank algorithms were not implemented as primary models because the leakage-clean pairwise training dataset contains only a small number of informative groups and effective unordered comparisons. Under this data regime, higher-capacity ranking models would not address the central bottleneck, which is the limited amount of diverse within-group comparative supervision. The hybrid pairwise formulation was therefore used as a transparent diagnostic of whether the available pairwise signal adds ranking value beyond the global model.

Text S6. Pairwise Dataset Structure and Statistics

The leakage-clean pairwise dataset used in this study was constructed exclusively from informative (DOI, analyte) groups identified within the training split of the final rebuilt pipeline. No pairwise samples were constructed from the full dataset or from the held-out test set.

In the final leakage-clean training set, 74 directional pairwise samples were generated from 11 informative groups, corresponding to 37 effective unordered comparisons under bidirectional augmentation. The number of directional pairwise samples per group ranged from 4 to 24, with a median of 4.

Although the nominal number of directional samples is non-zero, the effective diversity of the pairwise dataset remains limited. All pairwise comparisons are generated within a small number of groups, leading to repeated reuse of the same candidate systems and substantial within-group dependency. As a result, the pairwise dataset is highly structured and should not be interpreted as a large set of independent preference observations.

Importantly, these training-only pairwise statistics should not be conflated with the full-dataset informative-group count reported in Section 3.1. The full-dataset count describes the overall scarcity of informative competition across the reconstructed literature-derived dataset, whereas the pairwise dataset reported here reflects only the subset of informative groups available after split-first reconstruction and training-only pairwise construction.

Taken together, these characteristics indicate that the leakage-clean pairwise training dataset is small, redundant, and underpowered for drawing high-confidence conclusions about transferable preference learning across studies. Therefore, the pairwise component should be interpreted as a diagnostic probe of whether the available comparative supervision provides additional ranking signal, rather than as a definitive test of pairwise learning in general.

Table S5. Hyperparameter settings used for model training. Conservative configurations were selected to reduce overfitting given the limited size and structure of the dataset.

Model	Parameter	Value

Logistic Regression (l2)	penalty	l2
Logistic Regression (l2)	C	1
Logistic Regression (l2)	class_weight	balanced
Logistic Regression (l2)	solver	lbfgs
Logistic Regression (l2)	max_iter	5000
Logistic Regression (elasticnet)	penalty	elasticnet
Logistic Regression (elasticnet)	l1_ratio	0.5
Logistic Regression (elasticnet)	C	0.5
Logistic Regression (elasticnet)	class_weight	balanced
Logistic Regression (elasticnet)	solver	saga
Logistic Regression (elasticnet)	max_iter	8000
Random Forest	n_estimators	500
Random Forest	class_weight	balanced_subsample
Random Forest	random_state	42
XGBoost	n_estimators	400
XGBoost	max_depth	4
XGBoost	learning_rate	0.05
XGBoost	subsample	0.9
XGBoost	colsample_bytree	0.9
XGBoost	reg_lambda	1
XGBoost	objective	binary:logistic
XGBoost	eval_metric	logloss
Hybrid model	alpha_full	0.7
Hybrid model	alpha_pair	0.3

Table S6. Quantitative summary of the leakage-clean pairwise dataset structure

Metric	Value
Total directional pairwise samples	74
Unique informative groups (DOI, analyte)	11
Pair samples per group (median)	4
Pair samples per group (range)	4–24
Label distribution (preferred vs non-preferred)	balanced (by construction)
Pair construction method	bidirectional (i → j and j → i)
Effective unordered comparisons	37 (unique unordered pairs)
Redundancy structure	high (within-group reuse of candidates)
Informative vs total groups	11 training-informative groups used for pairwise construction; distinct from the 21 full-dataset informative groups reported in Section 3.1

Section S4 — Evaluation and Robustness Analyses

Text S7. Additional robustness analyses (Ablation and sensitivity)

- **Ablation study of the hybrid formulation**

Ablation analysis was performed to isolate the contributions of the full-model and pairwise components to ranking performance. Results show that the pairwise-only formulation performs substantially worse than the full-only model, and that the hybrid model does not improve over the

full component alone. These analyses were conducted on the comparable-group subset under leakage-clean conditions and are consistent with, but not identical to, the final holdout evaluation reported in Tables S7–S9. The results therefore support the conclusion that, under the present dataset structure and evaluation subset, the pairwise signal does not provide stable additional ranking value beyond the full-model component (Table S12).

- **Sensitivity of ranking performance to the hybrid weighting parameter**

Sensitivity analysis was performed by varying the hybrid weighting parameter α_{full} while evaluating ranking performance on comparable groups with at least two candidates. These analyses were conducted on the same restricted evaluation subset and should be interpreted as exploratory diagnostics rather than direct estimates of final holdout performance. The results indicate that the lack of hybrid improvement in this evaluation is not attributable to a specific weight choice alone, but is consistent with the limited and structurally constrained pairwise supervision available in the dataset (Table S13).

Text S8. Split-wise preprocessing, pairwise construction, and inference (pseudocode)

Below is a step-wise description of the leakage-clean pipeline applied in this study:

Step 1 — Grouped data splitting

- Partition dataset by DOI
- Assign all records from each DOI to either training or test set

Step 2 — Training-only preprocessing

- Fit preprocessing steps (missing-value filtering, variance filtering, collinearity filtering) using training data only
- Record selected feature set

Step 3 — Transformation of test data

- Apply the preprocessing transformations derived from training data to the test set
- No re-fitting is performed on test data

Step 4 — Pairwise dataset construction (training only)

- Identify informative (DOI, analyte) groups within the training set
- Construct pairwise samples only within these training groups
- Apply bidirectional construction (i → j and j → i)

Step 5 — Model training

- Train full (global classification) model on training records
- Train pairwise model on training pairwise dataset

Step 6 — Inference on held-out groups

- For each test (DOI, analyte) group:
    - Compute full-model probabilities for all candidates
    - Perform pairwise comparisons within the same test group
    - Aggregate pairwise scores at group level

Step 7 — Ranking and evaluation

- Rank candidates within each test group using model scores
- Compute group-level ranking metrics
- Perform restricted evaluation on comparable multi-candidate groups

This procedure ensures strict separation between training and test data and prevents leakage from outcome-derived or test-set information into model construction.

Table S7. Ranking performance on all groups and comparable multi-candidate groups under leakage-clean DOI-held-out evaluation.

Scope	N groups	Baseline Hit@1	Hybrid Hit@1	Baseline Hit@3	Hybrid Hit@3	Baseline MRR	Hybrid MRR	Hybrid better	Hybrid worse	Equal
All groups in predictions	119	0.983	0.975	0.983	0.983	0.987	0.980	0	3	116
Comparable groups ≥2	8	0.750	0.625	0.750	0.750	0.802	0.702	0	3	5
Comparable groups ≥3	6	0.667	0.500	0.667	0.667	0.736	0.603	0	3	3

Metrics were computed using the same group-level ranking logic as the sanity baseline comparison reported in Table S17. Comparable groups refer to held-out (DOI, analyte) groups containing at least two or at least three candidate DES systems.

Table S8. Ranking performance with uncertainty under leakage-clean DOI-held-out evaluation

Scope	N groups	Model	Hit@1	95% CI (Wilson)	MRR	95% CI (Bootstrap)
All groups	119	Baseline	0.983	0.9580–1.0000	0.987	0.9657–1.0000
All groups	119	Hybrid	0.975	0.9412–1.0000	0.980	0.9544–1.0000
Comparable groups ≥2	8	Baseline	0.750	0.3750–1.0000	0.802	0.5208–1.0000
Comparable groups ≥2	8	Hybrid	0.625	0.2500–1.0000	0.702	0.4280–1.0000
Comparable groups ≥3	6	Baseline	0.667	0.3292–1.0000	0.736	0.4224–1.0000
Comparable groups ≥3	6	Hybrid	0.500	0.1667–0.8333	0.603	0.2857–0.8889

Table S9. Group-wise statistical comparison.

Scope	n	Hybrid better	Hybrid worse	p-value
≥2 candidates	3	0	3	0.25
≥3 candidates	3	0	3	0.25

Text S9. Group-wise statistical comparison

An exact sign (binomial) test was applied to group-wise comparison outcomes derived from Table S9, with tied cases excluded.
For comparable groups with ≥2 candidates (n = 3), the hybrid model did not outperform the baseline in any group, and was worse in 3 groups (p = 0.25). The same result was observed for groups with ≥3 candidates (n = 3), where the hybrid model again showed 0 wins and 3 losses relative to the baseline (p = 0.25).
The results show that full-group ranking metrics are strongly influenced by trivial group composition, as both random and heuristic baselines achieve high apparent performance when most groups contain only one candidate or a single dominant optimum. In comparable multi-candidate groups, the baseline ML model outperforms both random ranking and the polarity-mismatch heuristic in Hit@1 and MRR, indicating that it captures non-trivial descriptor-level structure. However, the hybrid formulation does not provide stable additional benefit over the baseline. Detailed sanity-baseline results are reported in Table S17.

These results indicate that no statistically robust evidence supports hybrid superiority under the final leakage-clean evaluation. Instead, the observed pattern is consistent with the broader conclusion of this study: under the present sparse comparative supervision and limited comparable-group sample size, pairwise augmentation does not provide stable ranking benefit beyond the baseline model.

Table S10. Detailed cross-validation results across folds for baseline models. Metrics are reported for each fold to illustrate variability and robustness of model performance.

**Model**	**No. of features**	**ROC–AUC (mean)**	**ROC–AUC (SD)**	**PR–AUC (mean)**	**PR–AUC (SD)**	**MCC (mean)**	**MCC (SD)**	**Balanced accuracy (mean)**	**Balanced accuracy (SD)**
logreg	75	0.568881	0.168497	0.926342	0.05797	0.0238	0.109799	0.496437	0.097752
xgb	75	0.565677	0.100964	0.926287	0.047015	-0.00872	0.064539	0.484506	0.039424
rf	75	0.5845	0.113426	0.934727	0.044276	-0.04043	0.023996	0.491309	0.005982

Table S11. Impact of validation strategy on baseline model performance

**Validation Strategy**	**ROC–AUC**	**PR–AUC**	**MCC**	**Balanced Accuracy**
Random row-wise split	0.983	0.997	0.794	0.889
DOI-grouped split	0.802	0.868	0.629	0.779

Table S12. Ablation analysis of the hybrid model under leakage-clean DOI-held-out evaluation.

**Scope**	**Model**	**N groups**	**Hit@1**	**MRR**
All groups	Full-only	119	0.9748	0.9807
All groups	Pairwise-only	119	0.9412	0.9614
All groups	Hybrid	119	0.9580	0.9702
Comparable ≥2	Full-only	8	0.6250	0.7125

Comparable ≥2	Pairwise-only	8	0.1250	0.4262
Comparable ≥2	Hybrid	8	0.3750	0.5565
Comparable ≥3	Full-only	6	0.5000	0.6167
Comparable ≥3	Pairwise-only	6	0.1667	0.4016
Comparable ≥3	Hybrid	6	0.3333	0.4921

Table S13. Sensitivity of ranking performance to the hybrid weighting parameter (α) under comparable-group evaluation

**α_full**	**α_pair**	**N groups**	**Hit@1**	**MRR**
0.0	1.0	8	0.125	0.4262
0.3	0.7	8	0.125	0.4251
0.5	0.5	8	0.375	0.5543
0.7	0.3	8	0.375	0.5565
1.0	0.0	8	0.625	0.7125

Section S5 — Reproducibility Resources

Table S14. Software environment and libraries used for data processing and modeling.

**Component**	**Version**
Python	3.12.13
pandas	2.2.2
numpy	2.0.2
scikit-learn	1.6.1
matplotlib	3.10.0
RDKit	Not available in runtime environment (descriptors precomputed)
XGBoost	3.2.0

Table S15. Summary of the leakage-clean feature space used for modeling, grouped by descriptor family and functional role.

**Feature family**	**N features**	**Representative examples**	**Functional role**
Analyte descriptors	8	analyte_hba, analyte_hbd, analyte_heavyatoms	Physicochemical properties of the analyte
DES-level descriptors	16	des_hba_absdiff, des_hba_wmean, des_hbd_absdiff	Composition-weighted and contrast descriptors of the DES system

HBA descriptors	8	hba_hba, hba_hbd, hba_heavyatoms	Physicochemical properties of the hydrogen-bond acceptor
HBD descriptors	8	hbd_hba, hbd_hbd, hbd_heavyatoms	Physicochemical properties of the hydrogen-bond donor
DES-analyte interaction features	30	pair_hba_absdiff, pair_hba_product, pair_hba_ratio	Relative compatibility and interaction proxies between DES and analyte
Composition / ratio-derived features	5	ratio_hba_fraction, ratio_hba_part, ratio_hba_to_hbd	Descriptors derived from HBA/HBD composition and molar ratio

Table S16. Leakage-clean DOI-held-out classification performance of baseline and hybrid models.

Model	ROC-AUC	PR-AUC	MCC	Balanced Accuracy	Precision	Recall	F1-score
Baseline (LogReg) full clean	0.640648	0.749764	0.21471	0.604167	0.782258	0.808333	0.795082
Hybrid true pairwise clean	0.694444	0.787576	0.327805	0.675	0.833333	0.75	0.789474

Text S10. Sanity Baselines for Group-Level Ranking

Two sanity baselines were evaluated to contextualize group-level ranking performance under the leakage-clean DOI-held-out setting.

The first baseline consists of repeated random ranking within each (DOI, analyte) group, averaged over multiple independent permutations to estimate expected performance in the absence of learned structure. This baseline provides a lower-bound reference for evaluating whether model performance exceeds trivial ranking behavior.

The second baseline is a deterministic physicochemical heuristic based on analyte–DES polarity mismatch. Candidate DES systems were ranked by increasing absolute difference between analyte logP and DES-level weighted-mean logP. Lower mismatch was interpreted as better coarse polarity compatibility. This heuristic was selected because polarity matching is a simple, chemically interpretable, leakage-clean rule that can be computed directly from the descriptor space without using outcome-derived variables.

The polarity-mismatch heuristic is not intended to represent an optimized or universal DES-selection rule. Rather, it provides a conservative rule-based reference for assessing whether machine-learning models add value beyond a simple descriptor-level compatibility principle. Other chemically meaningful heuristics could be constructed from TPSA mismatch or hydrogen-bond complementarity. In this study, these descriptors were used as deterministic tie-breakers rather than as separate heuristic baselines, in order to keep the sanity check simple, transparent, and non-optimized.

Tie-breaking was performed deterministically using TPSA mismatch, hydrogen-bond balance mismatch, and DES system identifier to ensure reproducibility. Both sanity baselines were evaluated using the same group structure, ranking metrics (Hit@1, Hit@3, MRR), and restricted comparable-group subsets used for the primary model comparison.

The results show that full-group ranking metrics are strongly influenced by trivial group composition, as both random and heuristic baselines achieve high apparent performance when most groups contain only one candidate or a single dominant optimum. In comparable multi-candidate groups, the baseline ML model outperforms both random ranking and the polarity-mismatch heuristic in Hit@1 and MRR,

indicating that it captures non-trivial descriptor-level structure. However, the hybrid formulation does not provide stable additional benefit over the baseline. These results support the interpretation that meaningful recommendation assessment requires restricted comparable-group evaluation and comparison against simple sanity baselines.

Text S11. Alternative target definitions and ranking-model scope

Several alternative target formulations were considered conceptually but were not used as primary targets in this study.

Top-k labels would assign multiple candidates within a group as positive. This may reduce apparent class imbalance when groups contain several similarly performing systems, but it does not solve the scarcity of informative competition when most groups contain only one candidate or a single dominant optimum.

Recovery-threshold labels would define positive systems based on a fixed recovery margin relative to the best-performing DES. This approach may be useful in designed experiments with standardized conditions, but in literature-derived datasets recovery values are affected by study-specific matrix, pH, water content, extraction time, temperature, ionic strength, and reporting practices. A universal threshold could therefore introduce artificial comparability across heterogeneous studies.

Direct recovery regression would model recovery as a continuous outcome. This formulation addresses a different task from within-group recommendation and requires more complete and standardized experimental metadata than are available across the present dataset.

Pairwise ΔRecovery-based labels are conceptually aligned with ranking, but their reliability depends on the availability of sufficient within-group comparisons with meaningful recovery differences. The present leakage-clean training split contains only a small number of informative groups, limiting the robustness of such a formulation.

For these reasons, the present study used the group-relative is_optimal target as the primary operational target. This choice reflects the best observed DES system within each study-specific candidate set and is aligned with the recommendation question that can be most consistently reconstructed from the literature-derived data. The conclusions should therefore be interpreted under this target definition and should not be generalized to datasets with richer, designed, or standardized target structures.

Similarly, advanced learning-to-rank algorithms such as LambdaMART, RankNet, neural rankers, or listwise ranking models were not used as primary methods. These approaches generally require larger and more diverse query-group or preference datasets. In the present dataset, the primary limitation is not ranking-model capacity but the limited amount of leakage-clean comparative supervision.

Text S12. Suggested AI-ready reporting checklist for DES extraction studies

To improve the utility of future DES extraction studies for machine-learning-based recommendation, experimental reports should preserve the full candidate set and provide structured metadata for each tested DES–analyte combination. The following information is recommended for AI-ready reporting:

• Study identifiers: DOI, title, year, and source table or figure.

• Analyte information: analyte name, normalized name, chemical identifiers, SMILES when available, and analyte family.

• DES composition: HBA identity, HBD identity, molar ratio, water content if applicable, and chemical identifiers for each component.

• Candidate-set structure: all DES systems tested for the same analyte, including optimal, near-optimal, low-performing, and rejected candidates.

• Experimental context: matrix, sample amount or volume, extraction technique, pH, ionic strength or salt addition, extraction time, temperature, DES amount or volume, and phase-separation conditions.

• Analytical performance: recovery, uncertainty or replicate information, enrichment factor, LOD/LOQ values with units, and whether values are analyte-specific or averaged.

• Provenance: exact table, figure, or Supporting Information location for each record.

• Comparability annotation: indication of which DES candidates were compared under identical experimental conditions.

This checklist is not intended to prescribe a single experimental design. Rather, it defines the minimum information needed to reconstruct meaningful candidate sets and evaluate whether ML models are learning decision-relevant ranking relationships rather than study-specific reporting artifacts.

Table S17. Sanity baseline comparison under leakage-clean DOI-held-out evaluation.

Scope	n-groups	Random Hit@1 (mean ± SD)	Random Hit@3 (mean ± SD)	Random MRR (mean ± SD)	Heuristic Hit@1	Heuristic Hit@3	Heuristic MRR	Baseline Hit@1	Baseline Hit@3	Baseline MRR	Hybrid Hit@1	Hybrid Hit@3	Hybrid MRR
All groups	119	0.951 ± 0.010	0.978 ± 0.008	0.967 ± 0.006	0.950	0.983	0.966	0.983	0.983	0.987	0.975	0.983	0.980
≥2 candidates	8	0.273 ± 0.147	0.674 ± 0.125	0.507 ± 0.095	0.250	0.750	0.489	0.750	0.750	0.802	0.625	0.750	0.702
≥3 candidates	6	0.193 ± 0.156	0.566 ± 0.167	0.423 ± 0.113	0.167	0.667	0.402	0.667	0.667	0.736	0.500	0.667	0.603

Random baseline metrics are reported as mean ± standard deviation across repeated permutations. The heuristic baseline is deterministic and based on analyte–DES polarity mismatch with fixed tie-breaking rules. Baseline and hybrid metrics were computed using the same group-level ranking logic as Table S7 to ensure direct comparability across model-based and sanity-baseline evaluations.

Table S18. Dataset flow from literature search to final leakage-clean modeling table.

Stage	Count	Unit	Description
Scopus records identified	207	articles	Search using “deep eutectic solvents pesticides” in title, abstract, and keywords, limited to research articles
Full texts obtained	197	articles	Full-text PDFs were obtained for 197 articles
Studies retained after relevance screening	132	studies	Studies relevant to DES-based pesticide determination or extraction and retained for experiment-level extraction
Initial experiment-level records extracted	956	records	Records generated by structured AI-assisted extraction before final cleaning
Studies represented in final modeling table	94	studies	Studies with sufficient experiment-level data, chemical identifiers, recovery values, and descriptor eligibility
Final modeling records	757	records	Records retained after cleaning, consistency filtering, and descriptor eligibility filtering
Final competitive groups	626	(DOI, analyte) groups	Local decision groups used for target definition and ranking evaluation

Table S19. Permutation feature importance of the leakage-clean baseline model.

Rank	Descriptor	Feature family	Permutation ROC–AUC decrease (mean ± SD)	Chemical interpretation
1	analyte_hba	Analyte descriptor	0.093 ± 0.016	Hydrogen-bond acceptor capacity of the analyte
2	hbd_logp	HBD descriptor	0.078 ± 0.022	Hydrophobicity/polarity of the hydrogen-bond donor
3	analyte_molwt	Analyte descriptor	0.069 ± 0.027	Molecular size of the analyte
4	pair_heavyatoms_product	DES–analyte interaction	0.059 ± 0.020	Size/dispersion proxy for DES–analyte interaction
5	des_hbd_absdiff	DES-level descriptor	0.055 ± 0.020	Internal contrast in hydrogen-bond donor capacity within the DES
6	pair_hba_ratio	DES–analyte interaction	0.049 ± 0.012	Relative H-bond acceptor contrast between analyte and DES
7	des_tpsa_wmean	DES-level descriptor	0.048 ± 0.017	DES-level polar surface area proxy
8	analyte_logp	Analyte descriptor	0.048 ± 0.012	Analyte polarity/hydrophobicity
9	pair_hba_absdiff	DES–analyte interaction	0.048 ± 0.011	H-bond acceptor mismatch between analyte and DES
10	pair_hbond_acceptor_donor_product	DES–analyte interaction	0.044 ± 0.013	Donor–acceptor complementarity between DES and analyte
11	hbd_tpsa	HBD descriptor	0.043 ± 0.012	Polar surface area of the hydrogen-bond donor
12	analyte_ringcount	Analyte descriptor	0.040 ± 0.022	Aromaticity/structural rigidity proxy of the analyte
13	hbd_heavyatoms	HBD descriptor	0.034 ± 0.016	Molecular size of the hydrogen-bond donor
14	pair_tpsa_match_binary	DES–analyte interaction	0.034 ± 0.010	Binary polar surface compatibility between DES and analyte
15	des_rotbonds_wmean	DES-level descriptor	0.033 ± 0.013	DES-level flexibility proxy
16	pair_hbond_complement_sum	DES–analyte interaction	0.031 ± 0.011	Combined donor–acceptor complementarity
17	pair_logp_product	DES–analyte interaction	0.027 ± 0.014	Joint polarity/hydrophobicity interaction proxy
18	pair_hbd_product	DES–analyte interaction	0.026 ± 0.021	Joint hydrogen-bond donor interaction proxy
19	pair_heavyatoms_ratio	DES–analyte interaction	0.025 ± 0.018	Relative size contrast between analyte and DES
20	pair_logp_match_binary	DES–analyte interaction	0.024 ± 0.012	Binary polarity compatibility between analyte and DES

Permutation importance was computed on the DOI-held-out test set using ROC–AUC as the scoring metric. Values represent the mean decrease in ROC–AUC after feature permutation across 50 repeats. Only the top 20 descriptors are shown. Descriptor interpretations are qualitative and should not be interpreted as causal chemical design rules.

Table S20. Paired ranking differences and effect sizes for baseline vs hybrid comparison.

Scope	n groups	Baseline Hit@1	Hybrid Hit@1	ΔHit@1	ΔHit@1 95% CI	Baseline MRR	Hybrid MRR	ΔMRR	ΔMRR 95% CI	dz (MRR)	Hybrid better	Hybrid worse	Equal	Sign-test p
All groups	119	0.983	0.975	-0.008	-0.025 to 0.000	0.987	0.980	-0.0067	-0.0195 to 0.0000	-0.108	0	3	116	0.25
Comparable ≥2	8	0.750	0.625	-0.125	-0.375 to 0.000	0.802	0.702	-0.0997	-0.2664 to -0.0030	-0.430	0	3	5	0.25
Comparable ≥3	6	0.667	0.500	-0.167	-0.500 to 0.000	0.736	0.603	-0.1329	-0.3512 to -0.0040	-0.502	0	3	3	0.25

Paired group-level differences were calculated as hybrid minus baseline. Confidence intervals for ΔHit@1 and ΔMRR were estimated by bootstrap resampling over groups. The paired standardized effect size d_z was computed for ΔMRR. Exact sign-test p-values were calculated after excluding tied groups.

Table S21. Error categories observed during manual validation and their potential downstream impact.

Error category	Audited field(s)	Description	Correction/check performed	Potential downstream impact
Component-name normalization	HBA, HBD	Non-standard, abbreviated, or inconsistent component names were encountered across source articles.	Component names were manually checked against the original article and Supporting Information and standardized to curated component names.	Incorrect DES identity; incorrect molecular descriptor assignment.
Molar-ratio parsing	Molar ratio	Fractional, non-integer, or visually encoded HBA:HBD ratios required manual checking.	Reported ratios were verified from source figures, tables, or optimization sections.	Incorrect DES composition; incorrect molar-ratio-weighted descriptors.
Multi-analyte assignment	Analyte, recovery	Some studies reported recoveries for several analytes in shared tables.	Analyte-specific rows or columns were manually cross-checked against source tables.	Incorrect (DOI, analyte) grouping; incorrect target assignment.
Recovery-value assignment	Recovery (%)	Recovery values were sometimes embedded in complex optimization or Supporting Information tables.	Recovery values were verified against the corresponding analyte and DES system.	Incorrect optimal label; altered group-level ranking.
Experimental-condition assignment	Time, temperature, extraction conditions	Some experimental conditions were visually encoded or reported in Supporting Information tables.	Conditions were checked against figures, captions, footnotes, and SI tables.	Weaker interpretation of study context and within-group comparability.
Unit interpretation	LOD, concentration units, recovery-related fields	Units were sometimes reported in table footnotes or Supporting Information rather than directly in the extracted field.	Units were manually verified against table notes and source sections.	Incorrect metadata interpretation; possible exclusion or misclassification of analytical-performance fields.
Provenance ambiguity	Source table, figure, or section	The same DES or analyte could appear across multiple optimization steps.	Table, figure, or section provenance was checked manually.	Reduced traceability; possible context mismatch.

Missing or unverifiable values	Any extracted field	Some values were not explicitly reported in the article or SI.	No inference was applied; values were retained as missing or excluded downstream when required.	Reduced final dataset size after cleaning and descriptor eligibility filtering.

Table S22. Representative examples of corrected extraction discrepancies identified during manual validation.

DOI	Field corrected	Extracted value before correction	Corrected value after validation	Source location checked	Error category	Potential downstream impact
10.1016/j.chroma.2021.462036	Extraction time	1 min	2 min	Main text, Section 3.2.2, Figure 3d	Experimental-condition assignment	Affects interpretation of extraction context and within-study comparability, although not used as a leakage-clean descriptor.
10.1016/j.talanta.2023.124831	DES HBA/HBD molar ratio for tetrabutylammonium bromide:terpineol	2:1	4:1	Main text, Section 3.3.2, Figure 4B	Molar-ratio parsing	Affects DES composition and molar-ratio-weighted descriptors.
10.1016/j.microc.2023.109226	DES HBA/HBD molar ratio for choline chloride:1-phenylethanol	1:0	1:6	Main text, Section 3.1.1, Figure 1	Molar-ratio parsing	Affects DES composition, descriptor calculation, and eligibility of the DES system.
10.1002/jssc.202100863	LOD unit	µg/L	µg/kg	Main text, Table 1; Table S2; Section 3.3	Unit interpretation	Affects interpretation of analytical-performance metadata; LOD was excluded from the leakage-clean feature space.
10.1002/jssc.202100863	Temperature	25 °C	80 °C	Supporting Information, Table S1	Experimental-condition assignment	Affects interpretation of DES preparation/experimental context; not used as a leakage-clean descriptor.
10.1016/j.microc.2022.107226	Recovery (%) of lindane	86.4	84.6	Supporting Information, Table S1	Recovery-value assignment	Can affect optimal-label assignment and group-level ranking if the corrected value changes within-group ordering.

Table S23. Chemical rationale of descriptor families used in the leakage-clean feature space.

Descriptor family	Representative descriptors	Chemical rationale	Main limitation
LogP / polarity descriptors	analyte_logp, des_logp_wmean, pair_logp_absdiff, pair_logp_match_binary	Approximate hydrophobicity and polarity matching between pesticide analytes and DES systems	Does not capture phase behavior, activity coefficients, or solvent microstructure
TPSA descriptors	analyte_tpsa, des_tpsa_wmean, pair_tpsa_absdiff, pair_tpsa_match_binary	Approximate polar surface compatibility and polar interaction potential	Does not capture explicit solvation or local hydrogen-bond networks
Hydrogen-bond descriptors	analyte_hba, analyte_hbd, des_hba_wmean, des_hbd_wmean, pair_hbond_complement_sum	Approximate donor–acceptor complementarity between analyte and DES components	Does not represent directional or dynamic H-bond networks
Molecular size descriptors	analyte_molwt, des_molwt_wmean, pair_heavyatoms_product, pair_molwt_absdiff	Approximate molecular size, dispersion-related interactions, and steric compatibility	Does not encode molecular shape or conformer-specific interactions
Structural flexibility descriptors	rotatable bonds, ring count	Approximate rigidity/flexibility and structural complexity	Coarse proxy; does not model conformational ensembles
DES composition descriptors	ratio_hba_fraction, ratio_hbd_fraction, ratio_hba_to_hbd	Preserve HBA/HBD composition and molar-ratio information	Limited to simplified compositional representation
DES–analyte transformations	absolute difference, ratio, product	Capture mismatch, proportional contrast, and simple interaction proxies	Descriptive transformations, not mechanistic interaction energies

These descriptors were selected as interpretable physicochemical proxies available across literature-derived records. They should not be interpreted as complete mechanistic descriptors of DES extraction performance.

Table S24. Alternative target formulations considered in relation to the present literature-derived DES extraction dataset.

Alternative formulation	Definition	Potential advantage	Main limitation in this dataset	Decision
Current group-relative is_optimal	Best observed DES within each (DOI, analyte) group	Matches study-specific recommendation structure recoverable from literature	Conditional on candidate set and reported context	Used as primary target
Top-k labeling	Label top k candidates within each group as positive	May reduce label sparsity in groups with several similar candidates	Many groups have one candidate or one dominant optimum; may create trivial positives in small groups	Discussed, not used
Recovery-threshold labeling	Label systems within a fixed recovery margin of the best as positive	Allows near-optimal systems to be treated as acceptable	Requires comparable recovery values across standardized conditions; difficult across heterogeneous studies	Discussed, not used
Direct recovery regression	Predict recovery as continuous outcome	Uses more information than binary labels	Recovery depends on matrix, pH, water content, extraction time, temperature, and other metadata not consistently available	Different task; not used
Pairwise ΔRecovery labeling	Define pairwise preferences from recovery differences	Closely aligned with ranking	Requires sufficient within-group comparisons and reliable recovery differences; too few informative groups	Discussed, not used

These formulations may be valuable in designed or more standardized datasets but were not used as primary targets because they require stronger assumptions about comparability or substantially richer within-group supervision.

Table S25. Minimum reporting elements recommended for AI-ready DES extraction datasets.

**Reporting element**	**Examples**	**Why it matters for ML recommendation**
Study provenance	DOI, title, source table/figure, SI location	Enables traceability and audit of extracted records
Analyte identity	Name, normalized name, SMILES, analyte family	Defines the decision context and descriptor calculation
DES identity	HBA, HBD, molar ratio, water content, component identifiers	Defines candidate systems and DES-level descriptors
Full candidate set	Optimal, near-optimal, low-performing, rejected DESs	Preserves within-group competition for ranking
Experimental conditions	Matrix, pH, ionic strength, extraction time, temperature, technique	Separates solvent effects from protocol and matrix effects
Analytical response	Recovery, replicate information, uncertainty, EF, LOD/LOQ units	Supports reliable target definition and comparison
Comparability metadata	Which candidates were evaluated under the same conditions	Identifies meaningful local ranking groups
Machine-readable format	Structured tables with one row per DES–analyte condition	Reduces extraction errors and supports reproducibility

These elements are intended to support reconstruction of comparable candidate sets and reliable evaluation of ML-based recommendation models.

Dataset S1

Dataset S1 and Code S1. Reproducibility Resources

Dataset S1 provides the curated experiment-level modeling table used in this study. It contains the final leakage-clean dataset after literature screening, data cleaning, chemical standardization, descriptor eligibility filtering, and target construction. The dataset includes 757 records from 94 source studies, organized into 626 (DOI, analyte) groups.

Code S1 provides the complete computational workflow for leakage-clean data processing, model training, and evaluation under the split-first, train-only paradigm used in this study. The workflow includes DOI-grouped data splitting, training-only preprocessing, leakage-clean feature filtering, construction of the pairwise dataset exclusively from training groups, model fitting, test-only inference, within-group ranking evaluation, uncertainty estimation, statistical comparison, sanity baseline evaluation, permutation feature-importance analysis, and paired ranking effect-size analysis.

The code reproduces the main results reported in the manuscript and Supporting Information, including classification metrics, group-level ranking performance, comparable-group evaluation, uncertainty estimates, exact sign-test summaries, sanity baseline comparisons, descriptor-importance results, and paired baseline–hybrid ranking differences. It ensures consistency of the implemented workflow with the leakage-control and evaluation principles described in Sections 2.4–2.8 and Text S8.

While the code enables reproducibility of the reported analyses, it should not be interpreted as a formal proof of the absence of all possible indirect dependencies beyond the explicitly controlled pipeline steps.

Dataset S1, Code S1, and the core reproducibility package—including audit outputs, ranking outputs, sanity baseline results, uncertainty/statistical outputs, feature-importance outputs, paired effect-size outputs, revised tables, and revised figures—are publicly archived at Zenodo: https://doi.org/10.5281/zenodo.21257665.

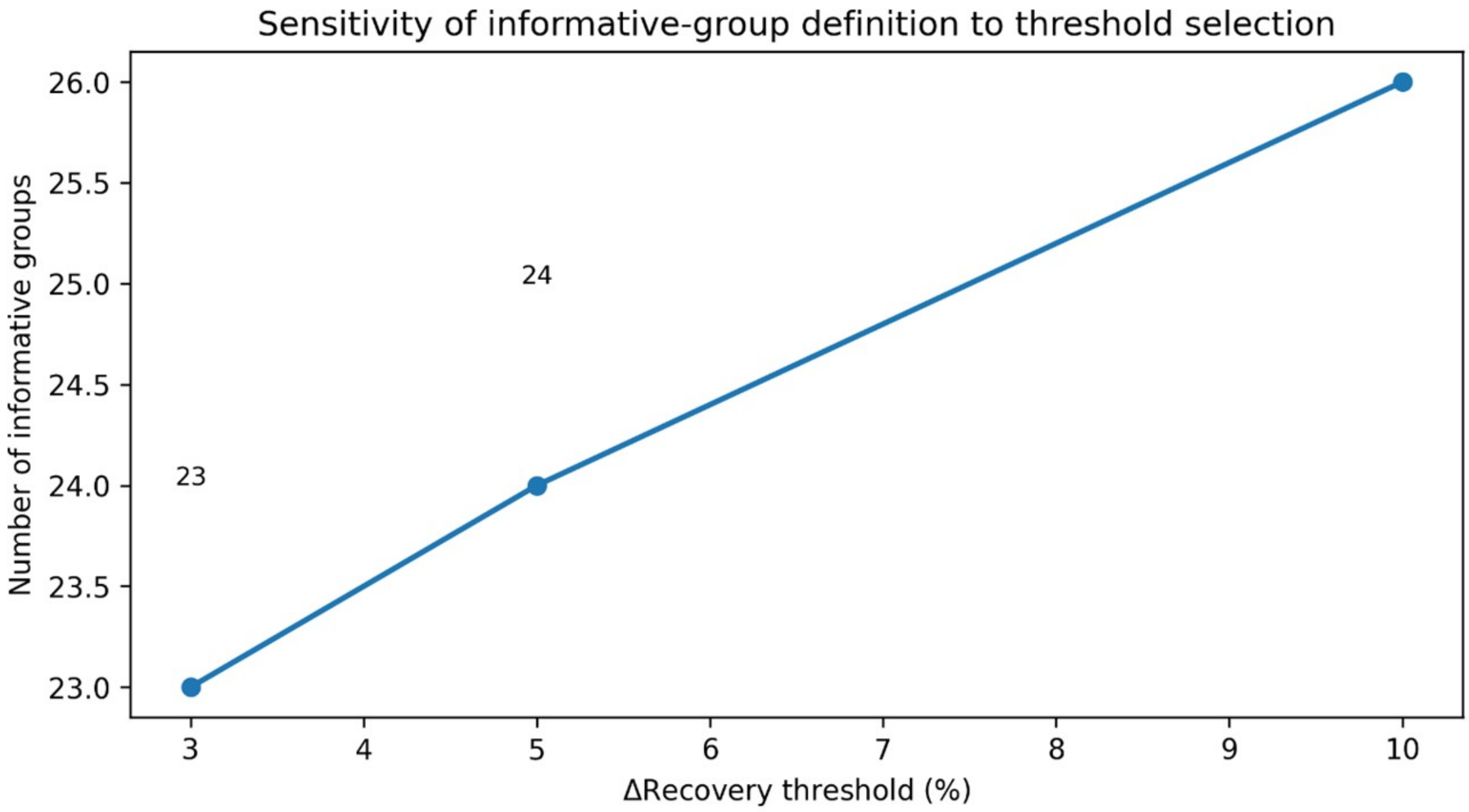

Figure S1. Sensitivity of the number of informative competitive groups to the ΔRecovery threshold used for group definition. The overall conclusion remains stable across reasonable threshold choices, indicating that the scarcity of informative groups is not an artifact of the 5% threshold.
